# Multifunctional Infrared Polarizer Based on Aligned Growth of High‐Density Boron Nitride Nanotubes

**DOI:** 10.1002/advs.202501908

**Published:** 2025-06-04

**Authors:** Ningqiang Shi, Xiaofei Xiao, Mingyu Zhang, Zhijun Li, Chuncheng Ban, Jinchang Meng, Nannan Shi, Mingyang Wu, Zebo Zheng, Ling Li

**Affiliations:** ^1^ MEMS Center Harbin Institute of Technology Harbin 150001 China; ^2^ Blackett Laboratory Physics Department Imperial College London London SW7 2AZ UK; ^3^ National Key Laboratory of Laser Spatial Information School of Integrated Circuits Harbin Institute of Technology Shenzhen 518055 China; ^4^ Guangdong Provincial Key Laboratory of Aerospace Communication and Networking Technology Harbin Institute of Technology Shenzhen 518055 China; ^5^ School of Electronic Engineering Heilongjiang University Harbin 150080 China; ^6^ State Key Lab Optoelect Mat & Technol Guangdong Prov Key Lab Display Mat & Technol Sch Elect & Informat Technol Sun Yat Sen University Guangzhou 510275 China

**Keywords:** boron nitride nanotubes, flexible polarizer, high‐density, infrared polarization dichroism, phonon polariton

## Abstract

Boron nitride nanotubes (BNNTs) are an emerging mid‐infrared optical material that exhibits significant potential in nanoscale resonators, lasers, detectors, and sensors due to the strong light‐matter interactions induced by phonon polaritons (PhPs). However, the large‐scale controllable synthesis and device applications of BNNTs still face huge challenges. In this study, a substrate‐pretreated and boron source dual‐catalyst infiltrated growth method, and a damage‐free purification method toward the high‐density and aligned BNNT (ABNNT) thin films is developed. The polarization sensitivity and linear dichroism characteristics of BNNTs with a maximum anisotropy ratio of 4.53 in the mid‐infrared and near‐infrared regions are comprehensively verified. The nanotube‐alignment induced permittivity anisotropy outside RB and the near‐isotropic PhP absorption within RB enable a wavelength‐selective polarizer and absorber throughout a broad infrared range. Uniquely, the BNNT polarizer exhibits excellent stability of polarization dichroism under severe bending conditions, demonstrating great potential in flexible and wearable optical devices. This work can initiate the utilization of high‐density BNNT thin films in quantum information processing and high‐resolution infrared imaging applications.

## Graphical abstract

This paper presents a novel method for the high‐density, aligned growth synthesis and purification of boron nitride nanotubes (BNNTs). The polarization sensitivity and linear dichroism characteristics of BNNTs in the near‐infrared and mid‐infrared bands are comprehensively verified.

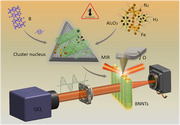

## 1 Introduction

Polarization of light is heart to quantum key distribution of quantum communication, and high‐contrast industrial and bio‐medical imaging. Polarized light detection and imaging are based on materials and structures with anisotropies.^[^ ^1^, ^2^, ^3^, ^4^ ^]^ Conventional metal‐grating‐based polarizers suffer from limitations in spectral bandwidth, mechanical flexibility, and structural stability, making them inadequate for meeting the increasingly dema nding performance requirements of modern optoelectronic chips. Low‐dimensional materials with decent optical anisotropy overcome these limitations and therefore receive increasing attention in these years.^[^ ^5^, ^6^, ^7^, ^8^ ^]^ For instance, black phosphorus forms a highly asymmetric band structure along different crystal axes in its folded honeycomb network, leading to anisotropic light absorption of 1.4 at 633 nm.^[^ ^9^ ^]^ With plasmonic structure integration, a high photocurrent ratio (armchair to zigzag) of 8.7 can be achieved at 1550 nm.^[^ ^10^ ^]^ Researchers have extensively found that the anisotropy of crystals arises from different lattice periodicities along distinct crystallographic directions, such as in *α*‐MoO_3_,^[^ ^11^ ^]^ Sb_2_Se_3_,^[^ ^12^ ^]^ ReS_2_,^[^ ^13^ ^]^ MoTe_2_,^[^ ^14^ ^]^ GeAs,^[^ ^15^ ^]^ and GeSe.^[^ ^16^ ^]^ However, their commercial applications are not only hindered by the lack of in‐plane optical anisotropy, but also limited by the narrow spectral response, poor mechanical stretchability, and high fragility. Comparatively, 1D nanotubes, such as boron nitride nanotubes (BNNTs) and carbon nanotubes (CNTs), exhibit remarkable optical anisotropy.^[^ ^17^ ^]^ Recently, Lynch et al.^[^ ^18^ ^]^ reported the significant and tunable optical dichroism of highly aligned semiconducting CNTs for large‐scale integration into electro‐optical systems. However, CNT‐based polarizers fail to deal with the mid‐infrared band due to the high reflection of light in this spectral range with their metallic and semiconducting electronic properties. Notably, exotic PhP phenomenon in BNNTs has been most recently unveiled in the mid‐infrared region with an extremely high quality factor.^[^ ^19^, ^20^, ^21^ ^]^ Strong resonant absorption induced by PhPs occurs within the Reststrahlen Band (RB),^[^ ^22^, ^23^ ^]^ but outside of which, the high aspect‐ratio of BNNTs leads to anisotropy of permittivity and polarized light transmittance. These unique optical properties of BNNTs could enable broadband and wavelength‐selective mid‐infrared polarization optics.

BNNTs are tubular materials composed of hexagonal lattices formed by alternating nitrogen (N) and boron (B) atoms.^[^ ^24^, ^25^, ^26^ ^]^ This unique structure endows BNNTs with not only excellent thermal stability and oxidation resistance, but also important applications in high‐quality‐factor optical resonators and highly confined light propagation.^[^ ^27^, ^28^, ^29^, ^30^, ^31^ ^]^ However, high‐quality growth of BNNTs is a fundamental obstacle to unleashing this potential toward practical devices.^[^ ^19^, ^32^ ^]^ By optimizing growth conditions, achieving highly oriented growth, and implementing proper post‐processing, the optical properties of BNNTs can be significantly enhanced.^[^ ^20^, ^33^ ^]^ Therefore, the development of mature BNNT growth techniques has become a critical task that urgently needs to be addressed in this field.

Chemical vapor deposition is considered one of the most promising approaches for synthesizing BNNTs.^[^ ^34^, ^35^, ^36^ ^]^ This method is characterized by strong controllability and wide applicability, but still faces challenges such as achieving directional growth, high‐density, high‐purity, and low yield. Recently reported techniques, including boronization,^[^ ^37^ ^]^ aluminum‐based active catalysis,^[^ ^38^ ^]^ and boron oxide deposition,^[^ ^39^ ^]^ exhibit controllable features but are still limited by issues such as non‐uniformity, chaotic growth, and high impurity content. Research on the directional growth of BNNTs is currently still limited. For instance, current studies mainly use CNT template methods^[^ ^40^, ^41^, ^42^ ^]^ and composite material stretching methods^[^ ^43^, ^44^ ^]^ to achieve the directional alignment of BNNTs. However, removing templates and avoiding defects have also become major challenges for these methods. Substrate pre‐treatment combined with catalytic growth design is an ideal solution to address these challenges. However, most BNNTs produced using current techniques contain impurities.^[^ ^45^, ^46^, ^47^, ^48^ ^]^ Therefore, there is an urgent need to develop an efficient and non‐destructive purification method. Amin et al.^[^ ^49^ ^]^ studied methods for detecting various impurities during the purification process, providing important technical references for assessing the purification quality of BNNTs.

This paper presents an innovative method to fabricate device‐level, high‐density, and well‐aligned BNNTs and demonstrates the utilization in broadband polarizer and wavelength‐selective absorber in the mid‐infrared band. A metal substrate pre‐treatment combined with a dual‐catalyst B‐source infiltration method is used to synthesize the scalable and dense ABNNTs, followed by an efficient, non‐destructive purification method. The transmission polarization response of ABNNTs is modeled with effective media theory and tested using polarization spectroscopy. The optical polarization response characteristics of BNNTs in near‐ to near‐to‐mid‐infrared range can be interpreted by the anisotropic permittivity and highly‐confined PhPs, yielding a maximum polarization anisotropy ratio of 4.53. The stability of the polarization response of BNNTs under extreme bending conditions is also verified. These findings provide valuable insights for the design of flexible polarizers, polarization filters, and wearable polarized light sensors.

## 2 Results and Discussion

**Figure 1:**
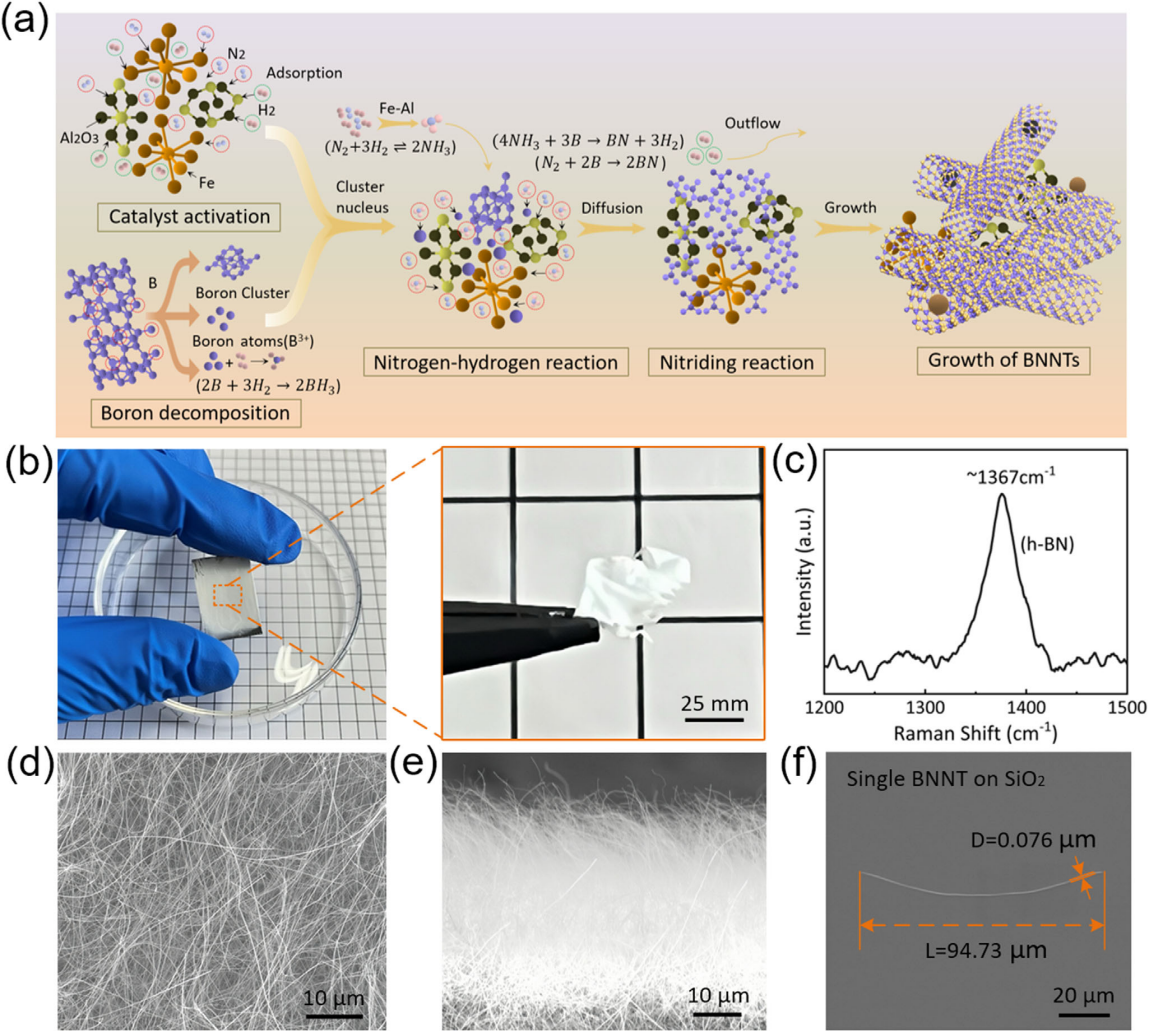
Growth principle and SEM images of densely packed BNNTs. a) Schematic diagram of highly dense growth of BNNTs. b) BNNTs grown on a metal substrate and the large‐area film after removal. c) Raman spectrum of the grown BNNTs. d,e) SEM images of BNNTs on the metal substrate. f) A single BNNT on a SiO2 substrate.

**Figure** **1**a illustrates the growth mechanism of BNNTs. Under high‐temperature conditions, boron and nitrogen sources generate reactive species, which diffuse and react around the catalyst to form BN. The synergistic catalysis of nanoscale Al_2_O_3_‐Fe is critical to the growth process: Al_2_O_3_ provides a stable support, while Fe acts as the active center to promote the reaction. Their cooperation enhances both the catalytic efficiency and the quality and uniformity of the resulting BNNTs. A chemical synthesis method was employed to prepare nanoscale Fe catalysts (see Figure S1, Supporting Information). Figure 1b shows BNNTs synthesized using the Al_2_O_3_–Fe dual‐catalyst system. The left image displays the uniformly distributed white BNNTs on the metal substrate (also present on the backside), while the right image shows a dense BNNT film after delamination. Figure 1c presents the Raman spectrum of BNNTs, featuring a prominent peak at 1367 cm^−1^ corresponding to the in‐plane E_2_g mode of h‐BN, indicating good crystallinity of the material.^[^ ^37^ ^]^ Figure 1d,e shows SEM images, revealing that the BNNTs grow densely with straight morphologies, without noticeable bending or entanglement. Figure 1f displays a single extracted BNNT with a diameter of ≈76 nm and a length of 94.73 µm.

To validate the critical role of catalysts in BNNT growth and demonstrate the advantages of the Al_2_O_3_‐Fe dual‐catalyst strategy proposed in this work, we conducted comparative experiments using various catalysts and combinations (Figure S2, Supporting Information). This approach also exhibited a high yield: under our experimental conditions, a single synthesis run produced highly dense BNNT films uniformly grown on both sides of four metal substrates (Figure S3, Supporting Information). Figure S4 (Supporting Information) presents the energy‐dispersive X‐ray spectroscopy (EDS) results of the BNNTs, further confirming their elemental composition, purity, and structural characteristics.

**Figure 2:**
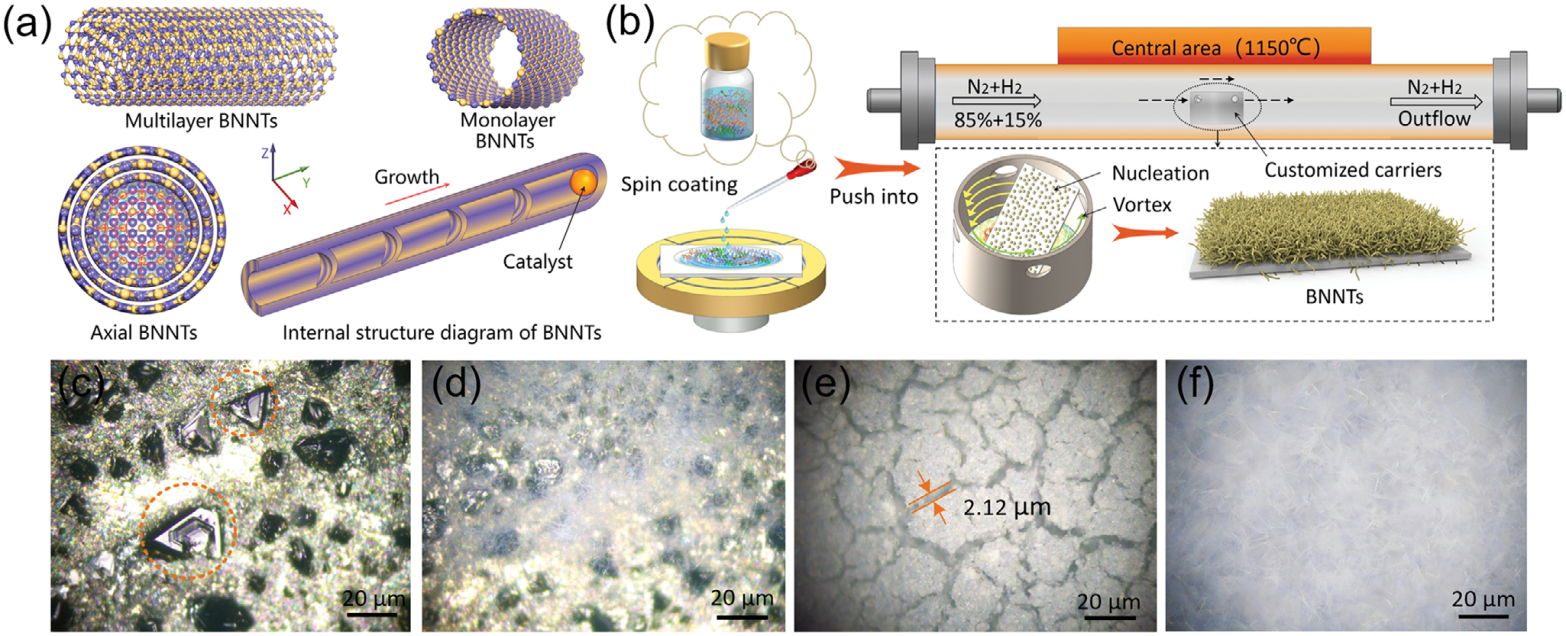
Growth of BNNTs on a metal substrate infiltrated with a B‐source and dual catalysts. a) Schematic diagram of the catalytic growth of BNNTs with different wall thicknesses. b) The process of BNNT growth in a tubular furnace, with the reaction zone temperature set at 1150 °C. c–f) Images of the four stages of BNNTs growth observed under optical microscopy. c) The nucleation stage of BNNTs. d) The stage where BNNT nuclei aggregate into clusters, accompanied by the growth of BNNTs. e) The hotbed formation stage of BNNTs, establishing the foundation for the subsequent high‐density growth stage. f) The high‐density growth stage of BNNTs.

**Figure** **2**a illustrates the multi‐walled and single‐walled structures of BNNTs along with a schematic of their catalyst‐assisted growth. As the growth is guided by metal catalysts, most catalyst particles remain at the tip ends of the BNNTs after growth. Figure 2b presents a schematic of the BNNT growth process inside a tube furnace. To ensure high purity and promote effective infiltration of the B‐source mixture, the metal substrates were electrochemically polished prior to growth (Section S2, Supporting Information). After polishing, the substrates were infiltrated with the B‐source solution and inclined inside a custom‐designed quartz boat. During the growth process, the mixed N₂/H₂ gas flow generated a vortex inside the quartz boat, which helped to overcome interfacial barriers between the N and B sources.

Figure 2c–f illustrates the four growth stages of BNNTs. Figure 2c corresponds to the first stage (nucleation), where nuclei with diverse morphologies are formed. Figure 2d shows the second stage (cluster aggregation), in which the nuclei increase in density and gradually aggregate into polygonal clusters, while BNNTs start to grow from the surface of the nuclei. Figure 2e depicts the third stage (formation of the foundational growth layer), also referred to as the “nursery” stage, where BNNTs intertwine to form island‐like structures. Figure 2f represents the fourth stage (high‐density growth), characterized by the dense growth of bright white BNNTs covering the entire substrate surface. To investigate the mechanism behind the high‐density synthesis of BNNTs, a thorough understanding of the nucleation process is essential. At elevated temperatures, the B source and dual catalysts jointly generate clusters of various morphologies, which adsorb N species to form numerous aggregated nucleation sites, laying the foundation for subsequent growth (Section S3, Supporting Information). In addition, by tuning the growth conditions, we experimentally captured the evolution of nuclei across different stages and the emergence of BNNTs from the nucleation sites (Section S4, Supporting Information).

**Figure 3:**
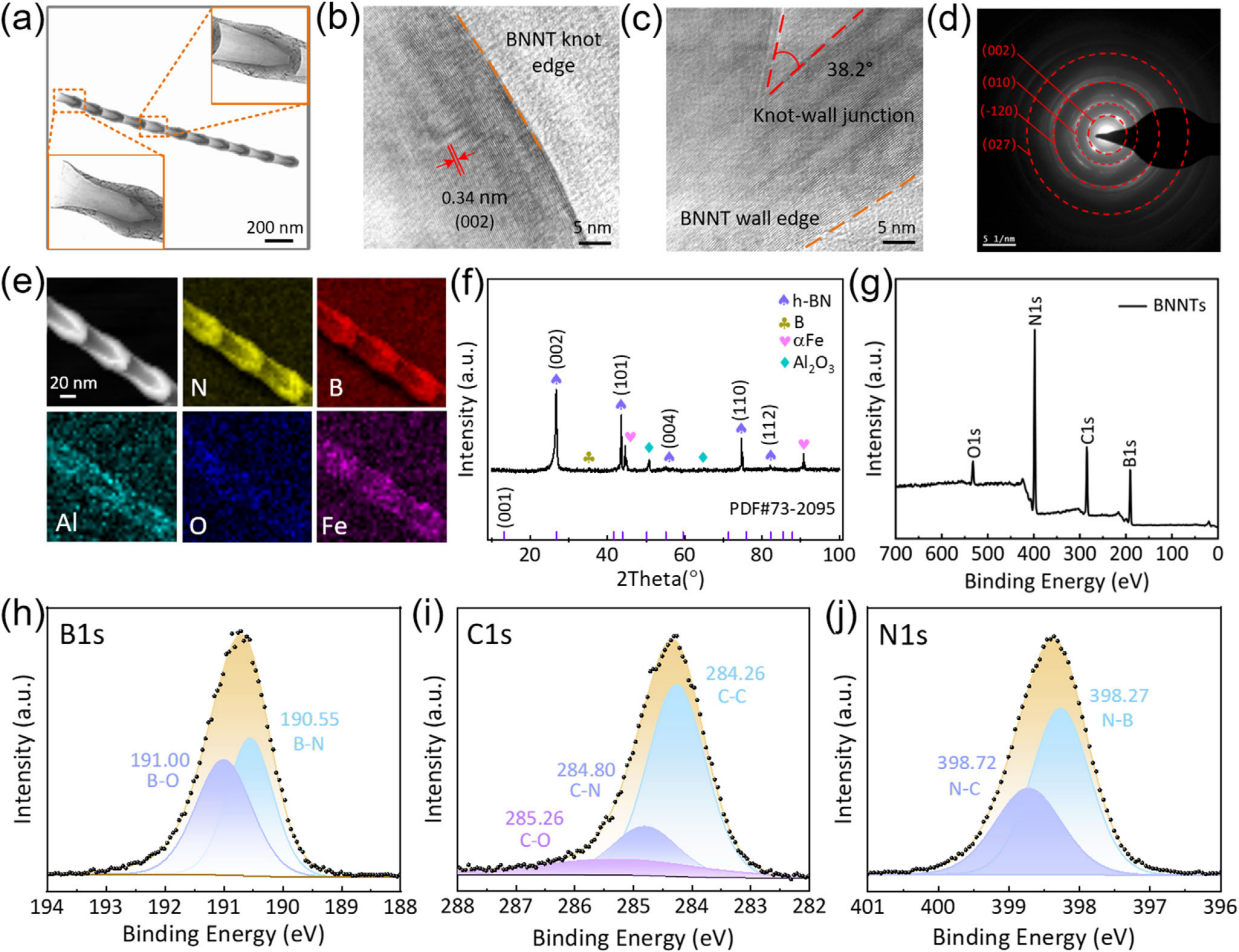
Characterization of the synthesized BNNTs samples. a) TEM image of a single BNNT. b) TEM images showing the edge and layered structure of the bamboo‐like end of BNNTs. c) SEM images showing the angle between the bamboo‐like end and the tube wall, and the tube wall edge of BNNTs. d) TEM diffraction pattern. e) HAADF‐STEM and elemental mapping images of BNNTs. f) XRD spectrum. g) XPS full spectrum. h) XPS spectrum of B1s. i) XPS spectrum of C1s. j) XPS spectrum of N1s.

**Figure** **3** systematically characterizes the microstructure, crystal features, and elemental composition of the synthesized BNNTs. Figure 3a shows a high‐resolution transmission electron microscope (TEM) image of a single BNNT, displaying its complete tubular structure. Figure 3b shows the layered edge structure at the bamboo‐joint end, with an interlayer spacing of 0.34 nm corresponding to the (002) plane, indicating a high degree of crystal order.^[^ ^50^ ^]^ Figure 3c shows a scanning electron microscope (SEM) image revealing the junction morphology between the bamboo‐joint and the tube wall, with an angle of 38.2°, reflecting the structural evolution during the growth process. Figure 3d presents a selected area electron diffraction (SAED) pattern, which identifies the (002), (010), (120), and (027) crystal planes, confirming the typical structure of h‐BN. Figure 3e shows a high‐angle annular dark‐field scanning transmission electron microscope (HAADF‐STEM) image and elemental mapping, indicating uniform distribution of B and N, with trace amounts of Fe and O detected, possibly originating from catalyst residues or surface adsorption.^[^ ^38^ ^]^ Figure 3f presents the X‐ray diffraction (XRD) pattern, where diffraction peaks corresponding to (001), (002), and (100) planes are observed, further confirming the crystallinity of BNNTs. Figure 3g–j shows X‐ray photoelectron spectroscopy (XPS) analysis, revealing that the binding energies of B1s (≈190.5 eV) and N1s (≈398.1 eV) are consistent with h‐BN, while the C1s peak (≈284.8 eV) may originate from environmental or test‐induced carbon contamination.

To verify the scalability of the BNNTs synthesis method we proposed, we achieved patterned growth of BNNTs and their synthesis on different substrates under the same preparation conditions (see Section S5, Supporting Information). In addition, we used thermal release tape (TRT) to peel BNNTs layer by layer and observed their length, density, and impurity distribution (see Section S6, Supporting Information). The results show that BNNTs on the “warm bed” surface have the longest average length (65 µm), while BNNTs inside the “warm bed” have the shortest average length (28 µm), and impurities are mainly concentrated in these areas. To improve the performance of BNNTs, it is necessary to employ efficient purification methods to remove unreacted B, catalyst residues, and impurities such as BN.^[^ ^45^ ^]^ We proposed a purification technique suitable for this method, effectively separating these impurities (see Section S7, Supporting Information). Furthermore, by improving the BNNTs preparation method (see the directional growth of BNNTs in the experimental section), combined with the pretreatment of the metal substrate and control of gas source conditions, we successfully synthesized directionally aligned BNNTs (Section S8, Supporting Information). This achievement lays the foundation for studying the multifunctional broadband infrared polarization‐sensitive properties of BNNTs.

**Figure 4:**
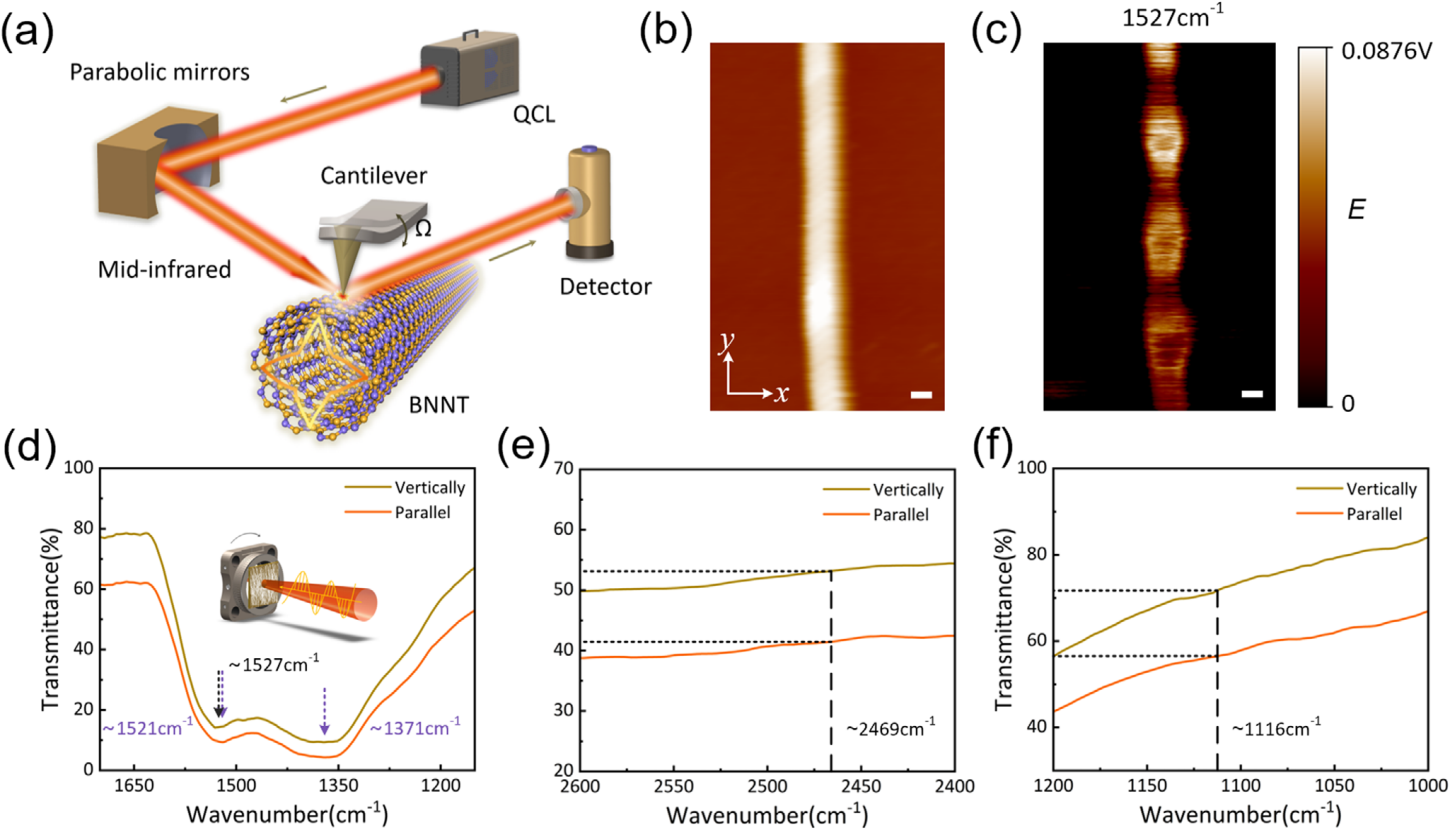
Direct observation of BNNTs PhPs modes and polarized spectral measurements. a) Schematic illustration of single BNNT characterization based on s‐SNOM. b) AFM morphology of BNNT on a flat gold substrate, with BNNT oriented longitudinally. c) Near‐field 2D distribution of the s‐SNOM signal at a frequency of 1527 cm^−1^. d–f) FTIR transmittance measurements at 1527, 2469, and 1116 cm^−1^ for parallel and perpendicular polarizations. The scale bar is 50 nm.

BNNTs exhibit high anisotropy typical of 1D nanotubes, with significant differences in the interaction of the electric field components of polarized light along the nanotube's axial and radial directions.^[^ ^39^ ^]^ To investigate the polarization‐sensitive properties of BNNTs in the mid‐infrared range, we first used scattering‐type scanning near‐field optical microscopy (s‐SNOM) to study the PhPs imaging of individual BNNTs. **Figure** **4**a shows a schematic of s‐SNOM characterization of the bamboo‐like structure of BNNTs and its interaction with PhPs propagation and response (for detailed principles, see Section S7, Supporting Information). To elucidate the interaction between mid‐infrared optics and BNNTs, we developed an optical dielectric response model and analyzed the variations in the effective dielectric tensors of BNNTs as a function of the filling factor (Figure S22, Supporting Information).

As shown in Figure 4b, the atomic force microscopy (AFM) image of a single BNNT (placed on gold‐coated SiO_2_) reveals a diameter of 84 nm (see the position profile in Figure S23 (Supporting Information), where the diameter is represented by the height of the BNNT). Figure 4c presents the near‐field image at a wavelength of 1527 cm^−1^ (6.55 µm), where the BNNT exhibits periodic bright and dark regions along its axial direction, indicating that the bamboo‐like structure induces polarization plasmon scattering and localization through lattice distortion. The periodicity of the stripes is directly related to the diameter of the BNNT and its bamboo‐like structure. Resonant enhancement of the local electromagnetic field is observed at the bamboo joints and the sidewalls of the nanotube (bright regions). According to the electromagnetic field intensity distribution extracted from the transverse radial direction of the BNNT (Figure S23, Supporting Information), the near‐field distribution of each cavity exhibits two peaks near the van der Waals sidewalls, which is consistent with the typical whispering‐gallery phonon‐polariton mode.^[^ ^51^ ^]^

We analyzed the far‐field spectral polarization response of BNNTs, simulated their polarization optical properties using the effective medium theory (EMT) (Figure S24, Supporting Information), and measured the spectral transmittance using polarized Fourier‐transform infrared spectroscopy (FTIR). Figure 4d–f shows the transmittance results at three wavenumbers (1527, 2469, and 1116 cm^−1^). Both the simulation and measurement results indicate that when the light polarization is parallel to the BNNTs axis (*p*‐polarization), the absorption (and reflection) rates are higher than in the perpendicular direction (*s*‐polarization). In the second Reststrahlen band (RB2, 1367–1610 cm^−1^), the spectral transmittance significantly decreases. The transmittance drop at 1371 and 1521 cm^−1^ (Figure 4d) is associated with the transverse and longitudinal optical modes of BNNTs, respectively. The proximity of 1527 to 1521 cm^−1^ further confirms that at 1527 cm^−1^, the local field enhancement effect of phonon‐polaritons reaches its peak, significantly increasing light absorption. Notably, the simulation results in Figure S24 (Supporting Information) indicate that when the light polarization is perpendicular to the nanotube axis, the TO mode is suppressed due to the constraints of the 1D crystal.

**Figure 5:**
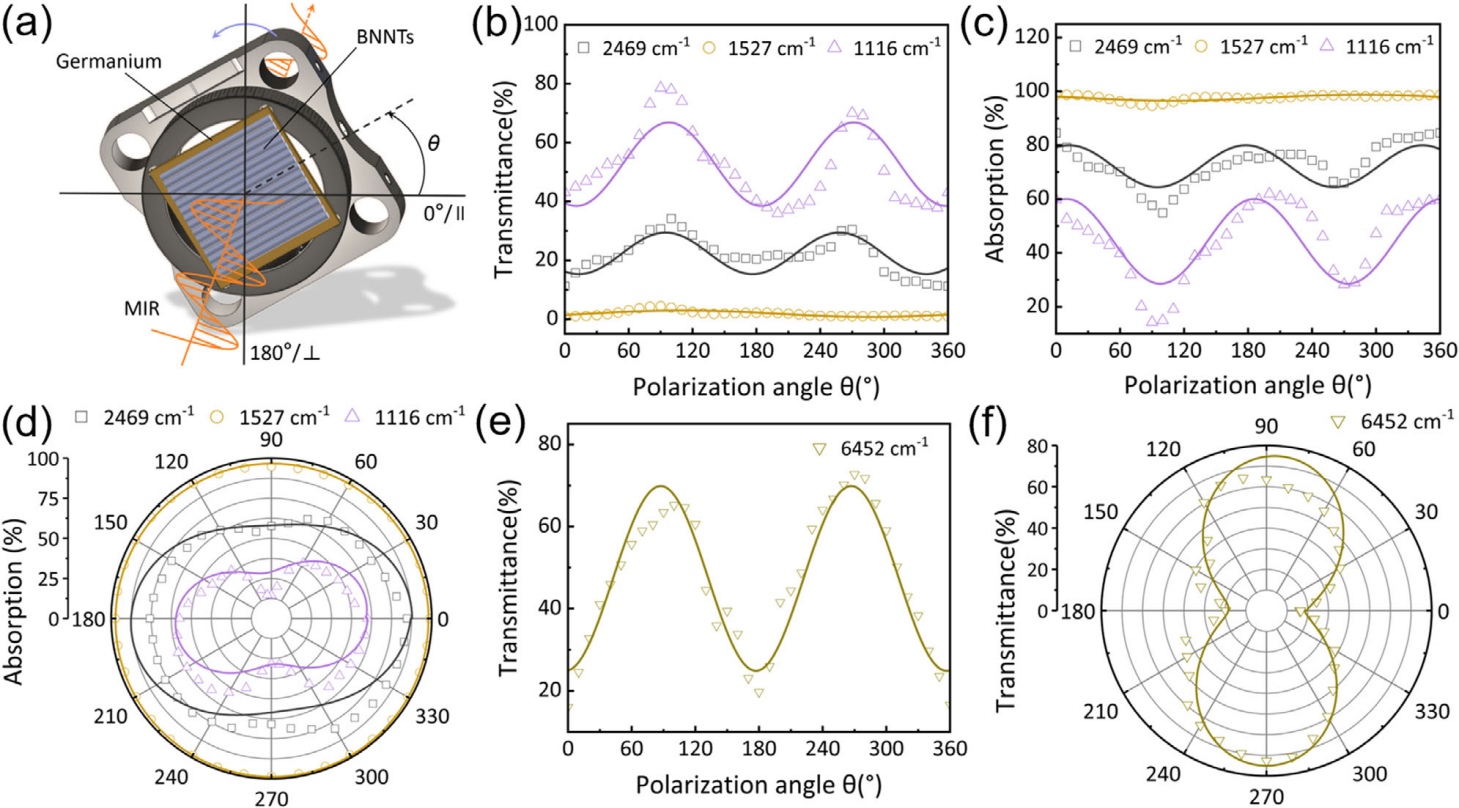
Infrared polarization‐sensitive properties of ABNNTs. a) Schematic diagram of polarization‐sensitive light transmission measurements. b) Polarization‐angle dependent transmission of ABNNTs under polarized light illumination at 1527, 2469, and 1116 cm^−1^, normalized and fitted with a sine function. c) Polarization‐angle dependent absorption of ABNNTs under illumination with three polarized light wavelengths. d) Polarization‐sensitive light absorption polar plots under three polarized light wavenumbers. e) Polarization‐angle dependent transmission of ABNNTs under 6452 cm^−1^ polarized light. f) Polarization‐sensitive light transmission polar plot under 6452 cm^−1^ polarized light.

Due to their unique lattice structure and geometry, BNNTs exhibit pronounced optical anisotropy. Well‐aligned BNNT (ABNNTs) films were transferred onto germanium substrates using an ethanol‐assisted method to investigate their polarization response at wavelengths of 4.05, 6.55, and 8.96 µm (corresponding to 2469, 1527, and 1116 cm^−1^, respectively). For convenience, wavenumbers are used in the following discussion. As illustrated in **Figure** **5**a, polarization‐resolved transmission measurements were conducted by rotating the sample to vary the polarization angle of the incident quantum cascade laser (QCL). Figure 5b shows the transmittance as a function of polarization angle at the three selected wavenumbers. The fitted curves exhibit sinusoidal variation with a 180° period: transmittance reaches a minimum when the polarization is parallel to the BNNT alignment and a maximum when it is perpendicular. Notably, the transmittance drops significantly at 1527 cm^−1^, indicating strong optical absorption in this band. As shown in Figure 4, this absorption is attributed to the excitation of phonon polaritons in the BNNTs.

Figure 5c shows the variation of absorption rate as a function of polarization angle. Based on reflectance measurements (Figure S25, Supporting Information), the calculated absorption of ABNNTs under parallel polarization is significantly higher than under perpendicular polarization, consistent with the results in Figures 4e and S24 (Supporting Information). As shown in Figure 5d, the polar plot of absorption exhibits a characteristic figure “8” pattern, indicating a pronounced anisotropic optical response. For incident polarized light at 2469, 1527, and 1116 cm^−1^, the maximum absorption rates are 84.56%, 98.75%, and 61.97%, respectively. The corresponding maximum dichroic ratios are 1.54, 1.04 ± 0.015, and 4.34. The small variation in absorption at 1527 cm^−1^ accounts for the reported uncertainty in this value. The degrees of polarization are 0.21, 0.02, and 0.63, respectively (see Experimental Section for calculation method). These results demonstrate that BNNTs exhibit strong selective linear dichroism and polarization sensitivity. Although absorption is high at 1527 cm^−1^, the low dichroic ratio and polarization degree suggest that this may be attributed to the intrinsic absorption characteristics of BNNTs in this RB. Overall, the polarization responses of BNNTs at 1527, 2469, and 1116 cm^−1^ are primarily governed by far‐field effects arising from crystalline anisotropy, while the contribution of near‐field phonon polariton excitation at 1527 cm^−1^ is relatively minor.

ABNNTs exhibit pronounced linear dichroism and polarization degree at 2469 and 1116 cm^−1^. Although the absorption at 1116 cm^−1^ is lower than that at 2469 cm^−1^, it shows more prominent dichroism and polarization. To gain deeper insight into the optical behavior of BNNTs, grazing‐incidence reflectance measurements were conducted at all three wavenumbers, with the results presented in Figure S26 (Supporting Information).

To verify the polarization properties of BNNTs in the optical communication band, we tested their polarization response at a near‐infrared wavelength of 1.55 µm (6452 cm^−1^). Figure 5e,d shows the polarization angle‐dependent transmission curves and polar plots of ABNNTs at this wavenumber. The results reveal that the transmission of vertically polarized light is significantly higher than that of parallel polarized light, with a linear dichroism of 4.53 and a polarization degree of 0.64, slightly higher than that in the mid‐infrared region. At 6452 cm^−1^, which is far from the RB region, the polarization dichroism is primarily due to dielectric anisotropy, while in the mid‐infrared region, it is limited by the PhPs effect. Although this study only selected representative wavenumbers for testing, the results indicate that ABNNTs exhibit broadband anisotropic optical response to infrared polarized light.

**Figure 6:**
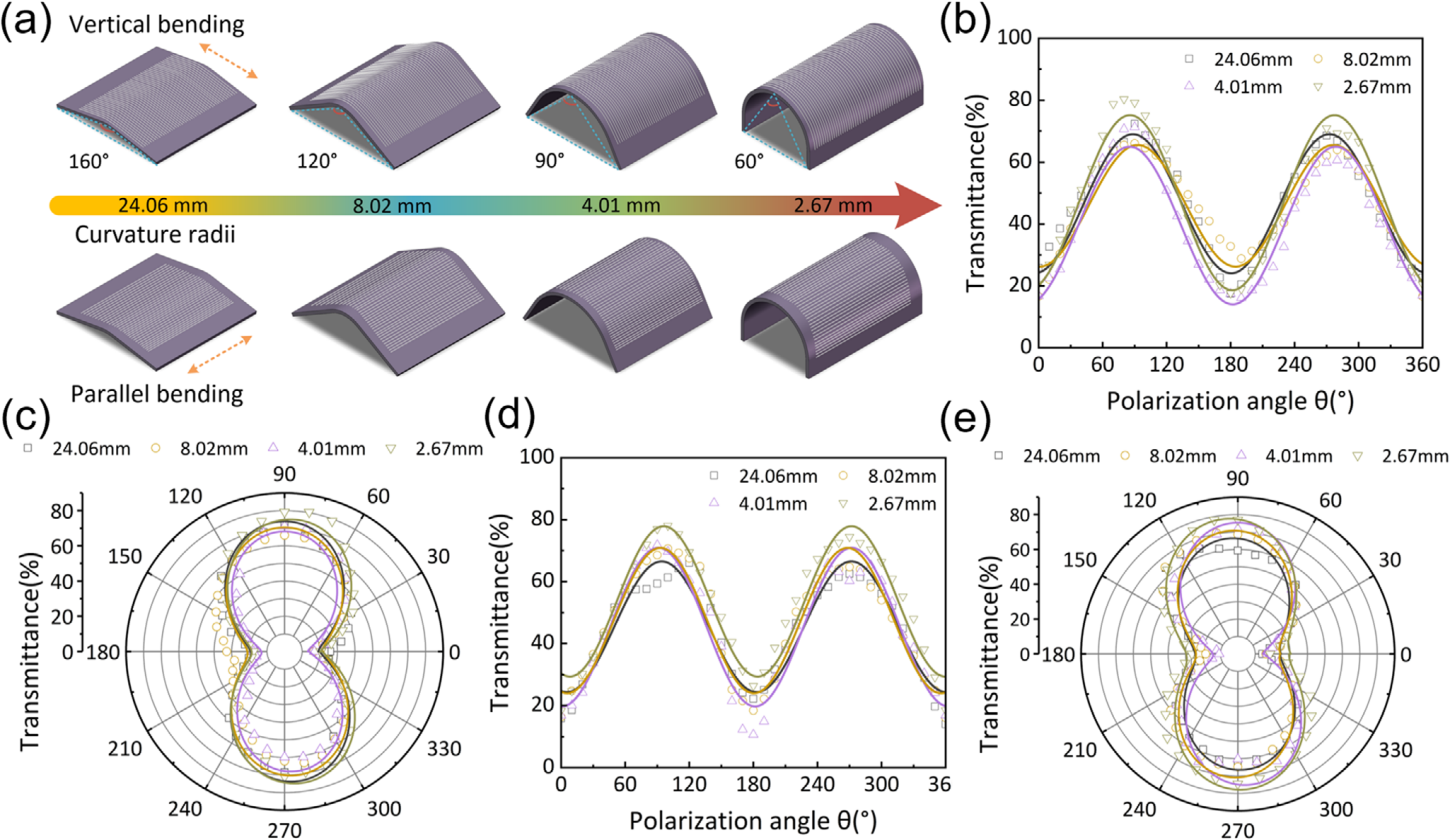
Optical transmission properties and polarization response of ABNNTs films under different bending states. a) Bending deformation states of ABNNTs films in vertical bending (upper row) and parallel bending (lower row) directions, and the corresponding curvature radii for different bending angles. b) The relationship between the polarization angle and the transmission of 6452 cm^−1^ polarized light under vertical bending with different curvature radii, normalized and fitted with a sine function. c) Polar plot of the relationship between the polarization angle and the transmittance under vertical bending with different curvature radii. d) The relationship between the polarization angle and the transmission of 6452 cm^−1^ polarized light under parallel bending with different curvature radii. e) Polar plot of the relationship between the polarization angle and the transmittance under parallel bending with different curvature radii.

To evaluate the stability of the polarization dichroism of BNNTs, we investigated the polarization response of ABNNTs under various bending conditions without using a mid‐infrared QCL, thereby eliminating the influence of phonon polaritons. A distributed feedback (DFB) laser operating at 6452 cm^−1^ was used to analyze the polarization sensitivity of ABNNT films under two bending modes: perpendicular (normal to the BNNT alignment direction) and parallel (along the alignment direction). To achieve flexible bending, the ABNNT film was transferred onto a polyvinyl alcohol (PVA) substrate, which offers both high infrared transmittance and good flexibility. As shown in **Figure** **6**a, we measured the polarization dichroism under different bending angles or radii of curvature (24.06, 8.02, 4.01, and 2.67 mm).

Figure 6b–e illustrates the variation in transmittance of polarized light at 6452 cm^−1^ as a function of polarization angle under four different radii of curvature. For perpendicular bending (Figure 6b,c), as the curvature radius decreases from 24.06  to 2.67 mm (indicating increased bending), the transmittance slightly increases by 7.87%, while the shape of the transmission curve remains nearly unchanged, suggesting that perpendicular bending has a minimal impact on the polarization characteristics of ABNNTs. In the case of parallel bending (Figure 6d,e), the transmittance also increases slightly by 7.64% with decreasing curvature radius. This difference may result from the varying tensile stress experienced by BNNT crystals under different bending orientations. Compared with current state‐of‐the‐art materials, the results of this work, as summarized in Table S1 (Supporting Information), demonstrate a significant advantage, highlighting the potential of ABNNTs for flexible applications such as wearable electronics and industrial thermal imaging.

## 3 Conclusion

In summary, this study proposes a novel method to achieve high‐density, high‐purity, and directional growth of BNNTs. PhPs modes are identified in BNNTs by s‐SNOM imaging at the mid‐infrared range. By virtue of the scalable ABNNT thin film, we systematically investigate the optical polarization response characteristics of BNNTs in the near‐infrared and mid‐infrared range for the first time, yielding a maximum polarization anisotropy ratio of 4.53. A trade‐off is observed between the measured polarization dichroism and the PhP‐mediated infrared absorption, exemplifying a multifunctional polarizer and absorber throughout a broadband infrared range. Minimal change of optical anisotropy under mechanical bending demonstrates the superiority of BNNTs compared to metallic gratings in maintaining stable polarization response. Our study provides innovative solutions for the application of BNNTs in polarization detectors, polarization filters, and flexible optical devices in a broadband infrared range.

## 4 Experimental Section

### Metal Substrate Treatment

The selected metal substrate is 304 stainless steel (X5CrNi18‐10). The cut metal substrates are ultrasonically cleaned with acetone, ethanol, and deionized water for 5–10 min. The cleaned metal substrate is used as the anode, while a metal substrate of the same size is used as the cathode, and both are placed parallel in the electrochemical polishing solution. The polishing voltage is set to 10–15 V, the current to 3–6 A, and the duration to 15–20 min. The polishing solution mainly consisted of deionized water, phosphoric acid (Aladdin, H_3_PO_4_, GR, ≥85 wt.% in H_2_O), ethanol (Aladdin, CH_3_CH_2_OH, AR, water ≤0.3%), isopropanol (Aladdin, AR, ≥99.7%), and urea (Aladdin, H_2_NCONH_2_, ≥99.5%). The volume ratio of these components is 9:10:5:2, with urea added at a concentration of 0.8–1.0 g per 100 mL of the total solution. The polished metal substrates are ultrasonically cleaned with deionized water and ethanol for 5–10 min and then dried with a nitrogen gun.

### Preparation of Nano Fe

Nano‐scale Fe is prepared by mixing and stirring ferrous sulfate heptahydrate (Aladdin, FeSO_4_·7H_2_O, ≥99.9% metals basis), sodium borohydride (Aladdin, NaBH_4_, 98%), and polyvinylpyrrolidone (Aladdin, PVP, (C_6_H_9_NO)n). A suitable amount of FeSO_4_·7H_2_O is dissolved in deionized water to prepare a 0.01–0.05 mol L^−1^ solution (50 mL), followed by sonication in a water bath for 5–10 min until the solvent is evenly dispersed. Similarly, an appropriate amount of NaBH_4_ is dissolved to prepare a 0.02–0.05 mol L^−1^ solution (50 mL), followed by sonication in a water bath for 5–10 min. 0.8–1.2 g of PVP is weighed and placed into a three‐neck flask, and the sonicated FeSO_4_·7H_2_O solution is then poured into the flask. The entire reaction setup is assembled and sealed to ensure airtightness. Ar is introduced to exclude air, and magnetic stirring is initiated. The reagents in the three‐neck flask react until the white polymer disappears. Subsequently, 50 mL of the NaBH_4_ solution is poured into a separatory funnel, and the valve is adjusted to allow the solution to drip into the three‐neck flask while stirring continuously until black material (Fe) is formed. The black material is washed 3–5 times with deionized water and 3–5 times with ethanol to obtain pure Fe, which is immediately transferred to an Ar glovebox for use.

### Preparation of BNNTs

Boron powder (Aladdin, B, 99.9% metals basis), aluminum oxide (Aladdin, nano Al_2_O_3_, 99.9% metals basis), and nano‐scale Fe are placed in an Ar glovebox and mixed with ethanol (2–4 mL) in a mass ratio of 12:4:1. The mixture is sonicated in a water bath for 10–20 min to achieve a homogeneous B‐source solution. The pre‐treated metal substrate is fixed onto a spin coater and coated on both sides with the B‐source solution at a speed of 300–500 r min^−1^ until the solution fully covers and penetrates the surface of the metal substrate. After spin coating, the substrate is immediately placed in a tubular furnace at 1150 °C using an alumina boat as the carrier. A mixed gas of N_2_ and H_2_ is introduced at a flow rate of 30–40 sccm for 30–60 min. The flow rate is then reduced to 5–10 sccm while cooling to room temperature, resulting in a dense BNNTs coating on the surface.

### Aligned Growth of BNNTs

The electrochemically polished metal substrate is mechanically polished using a polisher to create uniform grooves on the surface. The substrate is ultrasonically cleaned with acetone, ethanol, and deionized water for 5–10 min and then dried with a nitrogen gun. A clean brush is used to apply the B‐source solution along the direction of the grooves. The substrate is immediately suspended in an alumina boat and placed in a tubular furnace at 1150 °C for the reaction. A mixed gas of N_2_ and H_2_ is introduced at a flow rate of 20–25 sccm for 20–40 min. After the temperature decreased to 600 °C, the gas flow is adjusted to 5–10 sccm, and the furnace is cooled to room temperature to obtain surface‐aligned BNNTs.

### Purification of BNNTs

A clean metal scalpel is used to unidirectionally scrape BNNTs from the surface of the metal substrate at an angle of 10–30° (collecting only BNNTs above the base layer). Pure white BNNT films are obtained, with the film size determined by the scalpel width and scraping angle. The BNNTs are washed 3–5 times with deionized water and ethanol. The washed BNNTs are heated in a tubular furnace at 650 °C in air for 3 h, then the furnace is sealed, and a mixed gas of N_2_ and H_2_ is introduced for 1 h before cooling to room temperature for removal. The BNNTs are placed in a beaker, mixed with deionized water, and sonicated in a water bath for 20–30 min until uniformly dispersed. The BNNTs are heated in a water bath at 100 °C with deionized water and methanol 3–5 times, dried in an incubator, and leached in 3 M HCl solution (Aladdin, ACS, 37%) at 90 °C for 3 h. They are then washed 5–8 times with deionized water and ethanol, dried, and dispersed in a deoxycholic acid sodium salt (Aladdin, DOC, 98%) deionized water solution. The dispersion is sonicated in a water bath for 20–30 min until uniform, then filtered through a 1 µm pore membrane to obtain purified BNNTs.

### Material Characterization

The structural features of BNNTs were observed using scanning electron microscopy (SEM, Tescan, VEGA3 SBH), transmission electron microscopy (TEM, FEI, Talos f200x), and conventional optical microscopy (Nikon LV150N) with reflected light illumination and long working distance 20× and 50× bright‐field objectives. Tip‐enhanced Raman spectroscopy (TERS, Renishaw inVia‐Reflex) system, with laser excitation wavelengths of 225 and 514 nm, utilizes the tip‐enhanced effect to enhance the sensitivity and spatial resolution of the Raman signal, enabling the analysis of the chemical composition and optical properties of BNNTs at the nanoscale. X‐ray diffractometer (XRD, Panalytical, X'PERT) analyzes the diffraction peak intensities to determine the content of different chemical components and crystalline phases in the sample. X‐ray photoelectron spectroscopy (XPS, Thermo Fisher, Excalab 250Xi) and energy‐dispersive X‐ray spectroscopy (EDS, Thermo Fisher, NORAN system 7) are used to examine the elemental composition and quantitative information of the samples, with the C1s peak at 284.8 eV used as a reference. Fourier‐transform infrared spectroscopy (FTIR, Thermo Fisher, Nicolet iS50) is used to observe infrared absorption peaks and intensities in specific wavelength ranges to analyze the components and content of the samples. A simultaneous thermal analyzer (TG‐DSC, Netzsch, STA449F3, RT≈1500 °C) measures changes in sample mass during temperature variations, providing information on the evaporation, decomposition, oxidation, and reduction of volatile components.

### Infrared Polarization Sensitivity and s‐SNOM Experiments

The infrared sensitivity characteristics of the sample are measured using a NIR 1.55 µm distributed feedback laser (DFB, 0–50 mW, 1 MHz modulation frequency, free space) and a mid‐infrared quantum cascade laser (QCL, Alpes Lasers, Fabry‐Perot cavity, 4.05 µm wavelength, 0–350 mW, Distributive Feedback, 6.55 and 8.96 µm wavelength, 0–50 mW, three‐wavelength QCLs modulated at ≤50 kHz), along with a linear polarizer (Thorlabs, WP25H‐C‐CaF_2_ Holographic Wire Grid Polarizer, Ø25 mm, Mounted). A Fourier transform infrared spectrometer (Thermo Fisher, Nicolet iS50 Advanced FTIR), with a wavelength range of 2.5–25 µm, pre‐amplifier, external detectors, and external laser input/output interfaces, is used for spectral response measurements and can be used for polarization or vortex light characterization. Commercially available s‐SNOM is used for sample imaging. AFM is selected in tapping mode with a tip oscillation frequency of 285 kHz and an amplitude of approximately 50 nm. The near‐field intensity was defined as the square of the scattered field amplitude. An MCT detector (VIGO) converted the optical field into an electrical signal, which was demodulated at the third harmonic (3Ω) of the tip oscillation frequency using a lock‐in amplifier (Zurich).

### Calculation

During the infrared polarization‐sensitive measurements, the sample was mounted on a rotation stage with precise angular control, allowing measurements of the optical response at different polarization angles by rotating the sample. At each polarization angle, the transmittance (ω, 𝜃) and reflectance (ω, 𝜃) of the sample were measured. According to the principle of energy conservation, the absorptance (ω, 𝜃) is given by:

(1) A(ω,θ)=1−T(ω,θ)−R(ω,θ)

The dichroic ratio (DR) is defined as:

(2) $$DR=\frac{A⊥}{A∥}$$

Here, *A*∥ denotes the absorption for parallel polarization, and *A*⊥ denotes the absorption for perpendicular polarization.

The error range of the dichroic ratio is:

(3) $$\Delta DR=DR\sqrt{{\left(\frac{\Delta A∥}{A∥}\right)}^{2}+{\left(\frac{\Delta A⊥}{A⊥}\right)}^{2}}$$

In these experiments, the degree of polarization can be defined as the ratio of the difference in absorption or reflection between two orthogonal polarization directions (typically parallel and perpendicular to the alignment direction of the sample) to their total value:

(4) $$DOP=\frac{A∥-A⊥}{A∥+A⊥}$$

## Conflict of Interest

The authors declare no conflict of interest.

## Supporting information



Supporting Information

## Data Availability

The data that support the findings of this study are available from the corresponding author upon reasonable request.
