# Supplementary material for: Multifunctional Infrared Polarizer Based on Aligned Growth of High‐Density Boron Nitride Nanotubes
Source: Adv Sci (Weinh). 2025 Jun 4;12(30):e01908. doi: 10.1002/advs.202501908 (PMC12376592; doi:10.1002/advs.202501908)
Supplement: Supplementary file 1 — Supporting Information [file ADVS-12-e01908-s001.docx]

Supporting Information

**Multifunctional Infrared Polarizer based on Aligned Growth of High-Density Boron Nitride Nanotubes**

*Ningqiang Shi ^1^, Xiaofei Xiao^2^, Mingyu Zhang*, ^1, 3, 4^, Zhijun Li^3, 4^, Chuncheng Ban^5^, Jinchang Meng ^1^, Nannan Shi^3, 4^, Mingyang Wu^3, 4^, Zebo Zheng^6^, and Ling Li*^, 1^*

1. MEMS Center, Harbin Institute of Technology, Harbin 150001, China

2. Blackett Laboratory, Physics Department, Imperial College London, London SW7 2AZ, United Kingdom

3.National Key Laboratory of Laser Spatial Information, School of Integrated Circuits, Harbin Institute of Technology, Shenzhen 518055, China

4. Guangdong Provincial Key Laboratory of Aerospace Communication and Networking Technology, Harbin Institute of Technology, Shenzhen 518055, China

5. School of Electronic Engineering, Heilongjiang University, Harbin 150080, China

6. Sun Yat Sen Univ, State Key Lab Optoelect Mat & Technol, Guangdong Prov Key Lab Display Mat & Technol, Sch Elect & Informat Technol, Guangzhou 510275, China

* **Corresponding author.** E-mails: zhangmingyu@hit.edu.cn; linglimems@hit.edu.cn

**S1.** **Large-area dense growth and characterization of BNNTs**

Non-nanometric catalysts have a significant impact on the quality of BNNTs formation. In order to better cooperate with nanometric Al_2_O_3_ for the generation of high-quality BNNTs, we chemically prepare the storage-challenging nano-Fe. Figure S1 shows the schematic diagram of nano-Fe preparation. The detailed method can be found in the experimental section.


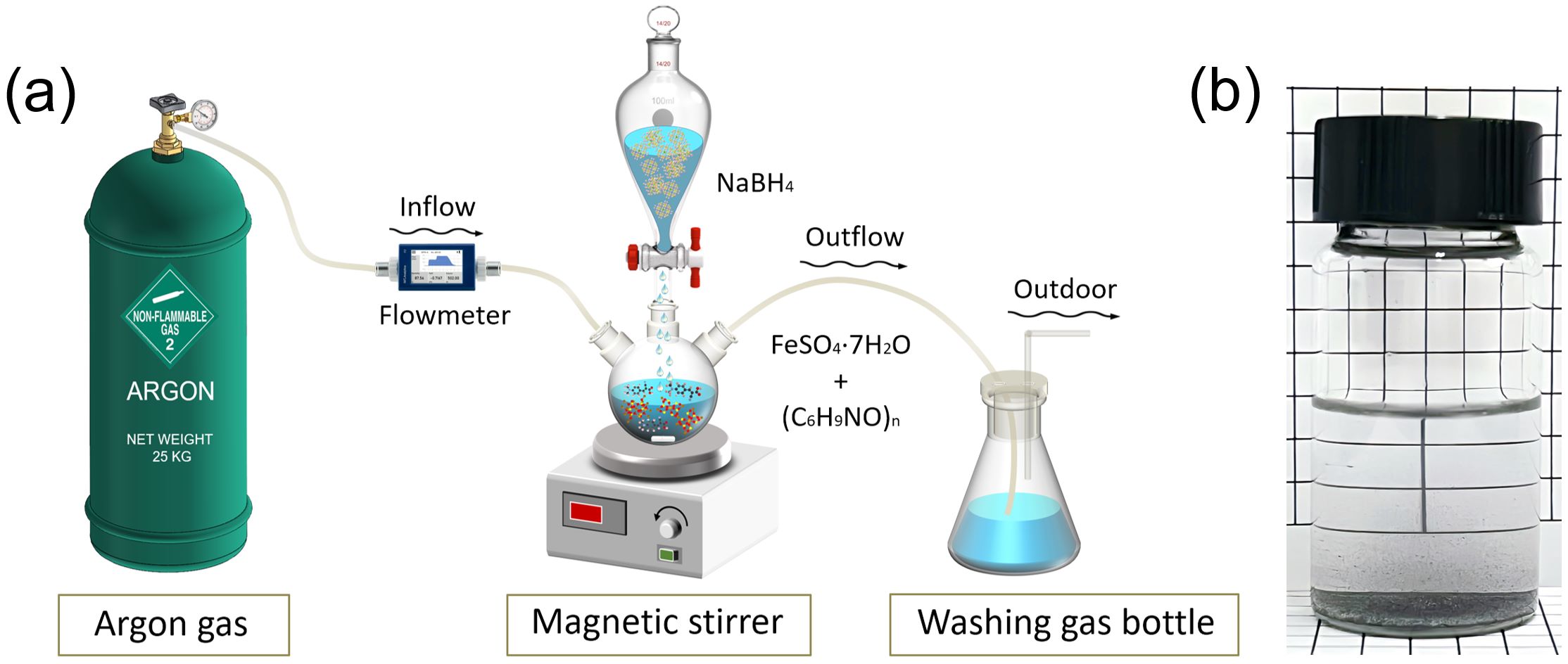


**Figure S1.** Schematic diagram and final product of nano-Fe preparation. a) Schematic diagram of nano-Fe preparation. b) Nano-Fe after washing.

To validate the advancement of this method using Al_2_O_3_ and Fe as main catalysts with the aid of metal substrate infiltration for BNNT synthesis, we tested Fe(NO_3_)_3_·9H_2_O, Al_2_O_3_, Fe, and their combinations as catalysts. As shown in Figure S2a, b, BNNTs synthesized with Fe(NO_3_)_3_·9H_2_O as the catalyst exhibit large and uneven diameters. In Figure S2, d, BNNTs synthesized using a combination of Fe(NO_3_)_3_·9H_2_O and Al_2_O_3_ as catalysts show significant improvements in diameter uniformity but exhibit pronounced twisting and bending. Figure S2e, f show BNNTs synthesized with nano-Fe as the catalyst, resulting in relatively uniform tube diameters, but the yield is sparse and limited. Figure S2e, f also show BNNTs synthesized using Al_2_O_3_ as the catalyst. Although densely packed BNNTs are obtained, the diameters remain non-uniform, with slight bending and twisting still observed. This shows a significant quality difference compared to the BNNTs synthesized with Al_2_O_3_-Fe dual catalysts, as illustrated in Figure 1d-f.


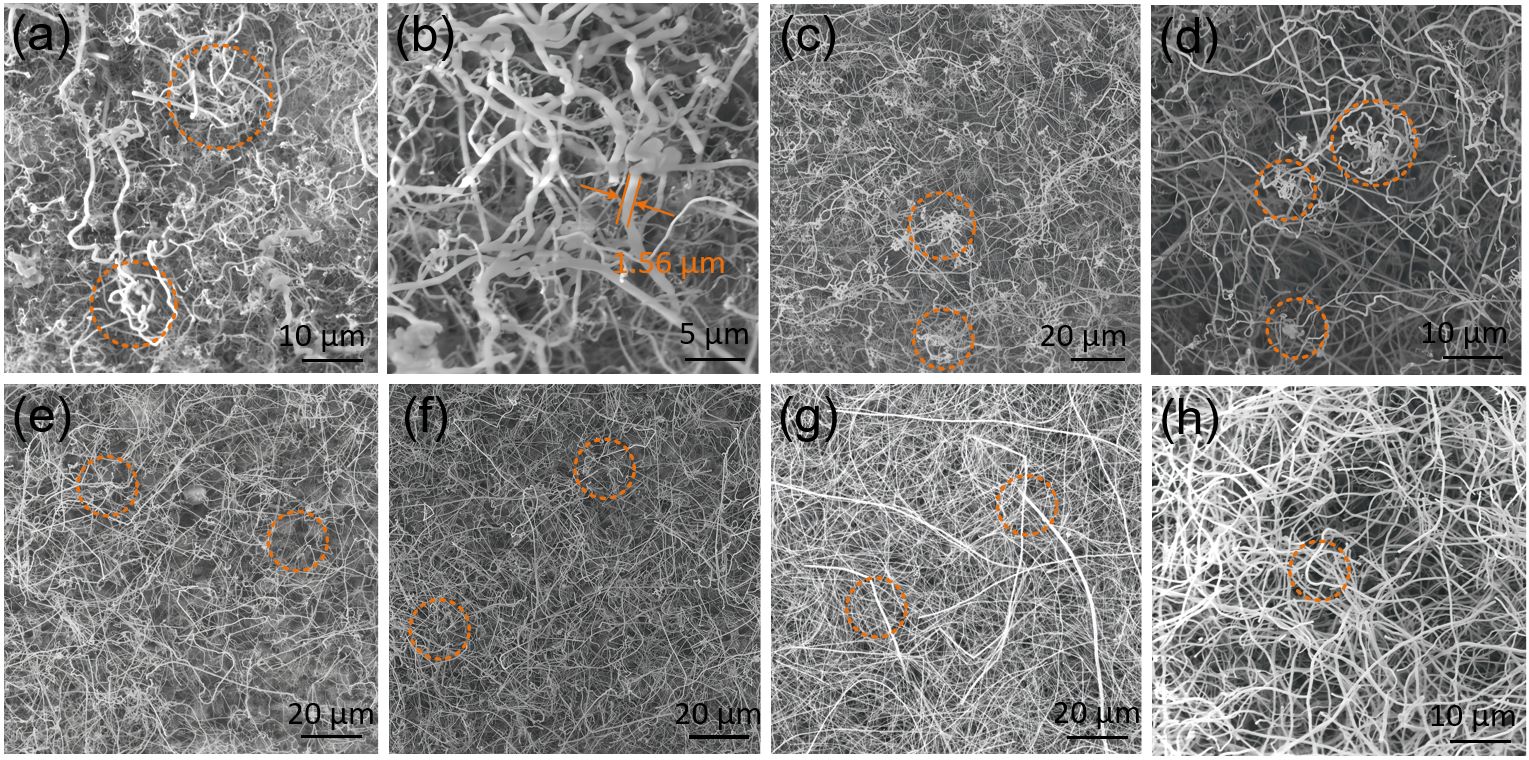


**Figure S2.** BNNTs synthesized with different catalysts and their combinations. a, b) Fe(NO_3_)_3_·9H_2_O as catalysts. c, d) Fe(NO_3_)_3_·9H_2_O and Al_2_O_3_ as catalysts. e, f) Fe as catalysts. g, h) Al_2_O_3_ as catalysts.

Through experimental comparisons, we found that BNNTs grown using the combination of nano-Al_2_O_3_ and Fe as catalysts exhibit higher quality. Observations reveal that nano- Al_2_O_3_ provides a stable high-surface-area substrate, disperses and regulates the auxiliary catalytic properties of Fe and the metal substrate, and increases active sites. Fe, serving as the primary active center of the catalyst, facilitates the reaction of boron and nitrogen as well as the nucleation and growth of BN. The synergistic effect of the two significantly enhances the nucleation density and growth quality of BNNTs. By controlling the properties and distribution of these catalysts, precise regulation of the BNNT growth process can be achieved.

Figure S3 shows the highly dense BNNTs synthesized in a single batch. Dense BNNTs are grown on both sides of each metal substrate. Due to equipment limitations, four samples, as shown in Figure S3, can be prepared in each batch. By further optimizing the equipment, this method has the potential to achieve higher yields and better-quality BNNT synthesis. Figure S4 shows the EDS spectrum of BNNTs on the metal substrate. The elemental composition of the synthesized BNNTs was analyzed, mainly consisting of B, N, O, Al, Mn, Cr, and Au. Among these, B, N, and Au were found in higher proportions. The presence of Au is for convenient SEM observation of the BNNT surface morphology, achieved by sputtering a 10 nm Au layer on the BNNT-grown metal substrate.


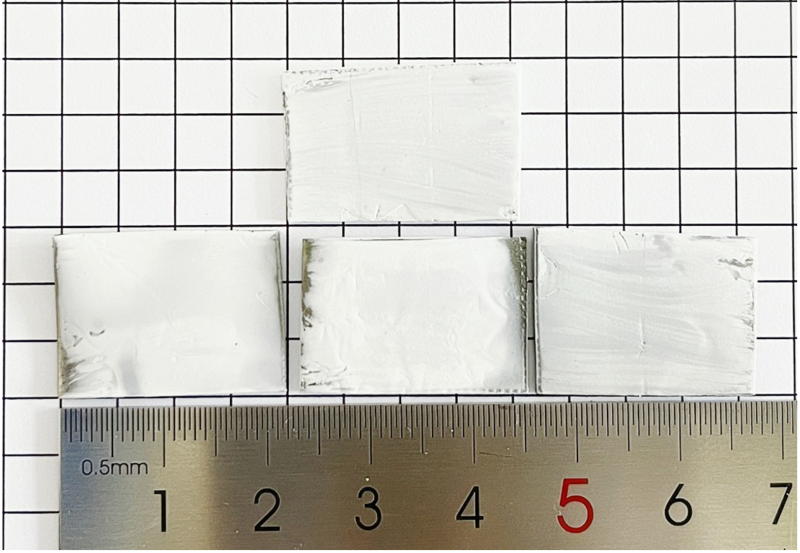


**Figure S3.** Highly dense BNNTs synthesized in a single batch.


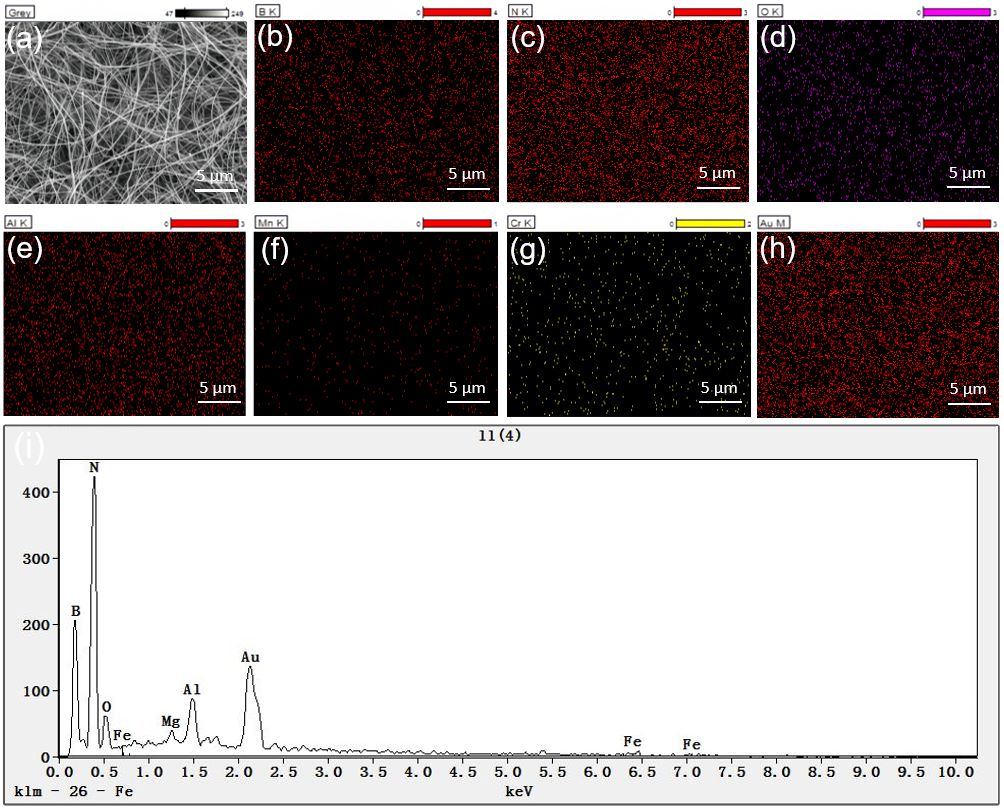


**Figure S4.** shows the EDS spectrum of BNNTs on the metal substrate. a) SEM image of the scanned area. b) B element. c) N element. d) O element. e) Al element. f) Mn element. g) Cr element. h) Au element. i) Elemental peak table.

**S2. Pretreatment of the Metal Substrate**

The quality of the substrate surface plays a crucial role in the initial nucleation and growth of materials such as BNNTs. The composition and surface structure of the underlying substrate largely determine the morphology, size, orientation, thickness, and overall quality of the overlying material. Therefore, substrate engineering, which involves purposefully selecting appropriate substrate materials or designing and pre-treating the substrate, becomes an important strategy for optimizing the material growth process.^[1]^

In the growth process of BNNTs, the pre-treatment of the metal substrate mainly includes cleaning and electrochemical polishing. Figure S5a shows a schematic of metal substrate cleaning and electrochemical polishing. Before electrochemical polishing, the metal substrate should be sequentially ultrasonically cleaned with acetone, ethanol, and deionized water, then dried with nitrogen or by heating at 100°C. Then, the metal substrate is used as the anode, where oxidation reactions occur during electrolysis, causing the metal surface to dissolve and generate metal ions (see reaction (1)-(3)). Since the current is mainly concentrated in the raised areas of the surface, the current density is higher, and the raised portions dissolve faster, leading to the gradual smoothing of the metal surface. At the same time, another metal substrate acts as the cathode, where a reduction reaction occurs (see reaction (4)). During the operation, it is necessary to ensure that the anode and cathode are placed in parallel in the electrolysis cell to optimize the polishing effect. After polishing, the sample should be removed from the electrolyte and ultrasonically cleaned with deionized water and ethanol to remove any residual electrolyte and ensure a clean surface.

Anode reactions:

$Fe\to Fe^{2+}+2e^{-}$ (1)

$Cr\to Cr^{3+}+3e^{-}$ (2)

$Ni\to Ni^{2+}+2e^{-}$ (3)

Cathode reaction:

$H^{+}+2e^{-}\to H_{2}\uparrow$ (4)


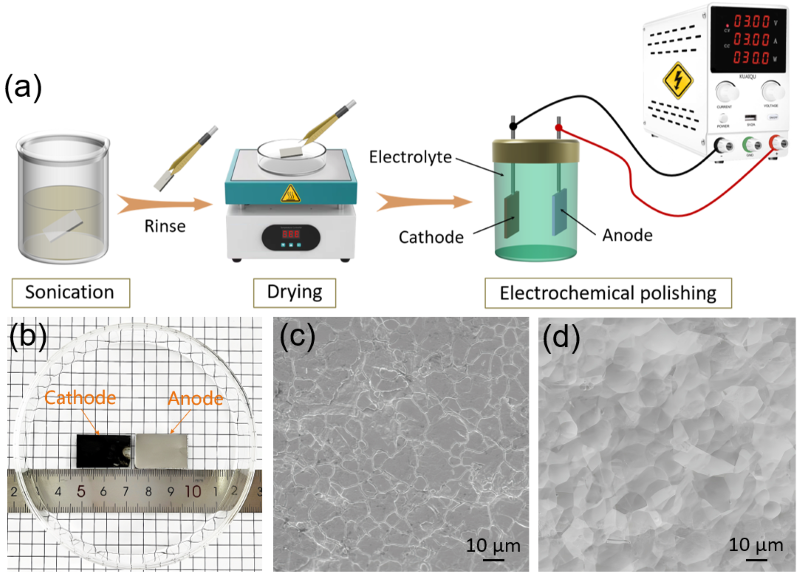


**Figure S5.** Metal substrate pre-treatment. a) Schematic diagram of metal substrate cleaning and electrochemical polishing. b) Anode and cathode of the metal substrate after electrochemical polishing. c) SEM image of the untreated metal substrate and d) SEM image of the pre-treated metal substrate.

As shown in Figure S5b, during electrochemical polishing, the cathode (left) surface is covered with a layer of metal impurities, which originate from materials stripped off the anode surface during polishing. After cleaning, the cathode can be reused multiple times. In contrast, the anode (right) surface remains smooth and shiny. As shown in Figure S5d, compared to the SEM image of the metal substrate before polishing (Figure S5c), the surface after electrochemical polishing has removed contaminants, defects, and oxide layers, showing a good surface condition, making it suitable as a substrate for BNNTs growth.

**S3. Initial Stage Nucleation of BNNTs**

To investigate the mechanism of high-density BNNT growth, it is necessary to gain an in-depth understanding of the nucleation process of BNNTs. Figure S6 illustrates the nucleation process of BNNTs. Figure S6a is a schematic diagram of the nucleation formation during the initial stage of BNNTs growth. Through repeated characterization of the BNNT growth process, we found that at a temperature of 1150°C, adsorption occurs between the precursors and catalysts, gradually leading to the formation of regular nuclei. The B-source, after being adsorbed onto the surface of nanoscale Al_2_O_3_ and Fe catalysts, decomposes into single boron atoms or small clusters. The N-source is similarly adsorbed and decomposed by the catalysts. Under high temperatures and the gas vortex inside the quartz boat, boron atoms and nitrogen atoms meet and combine to form BN allotropes structures. These BN allotropes structures gradually aggregate to form larger BN clusters, which further develop into well-defined nuclei with shapes such as triangles, quadrilaterals, triangular pyramids, and quadrangular pyramids. On the surfaces of these nuclei, the initial nucleation sites of BNNTs are progressively established. The nucleation sites on the nuclei's surfaces are also active sites of the catalysts, serving as key regions for BN allotropes formation. At these active sites, the adsorption and activation of B and N atoms enable rapid BN allotropes formation and surface diffusion, thereby promoting BNNT growth.


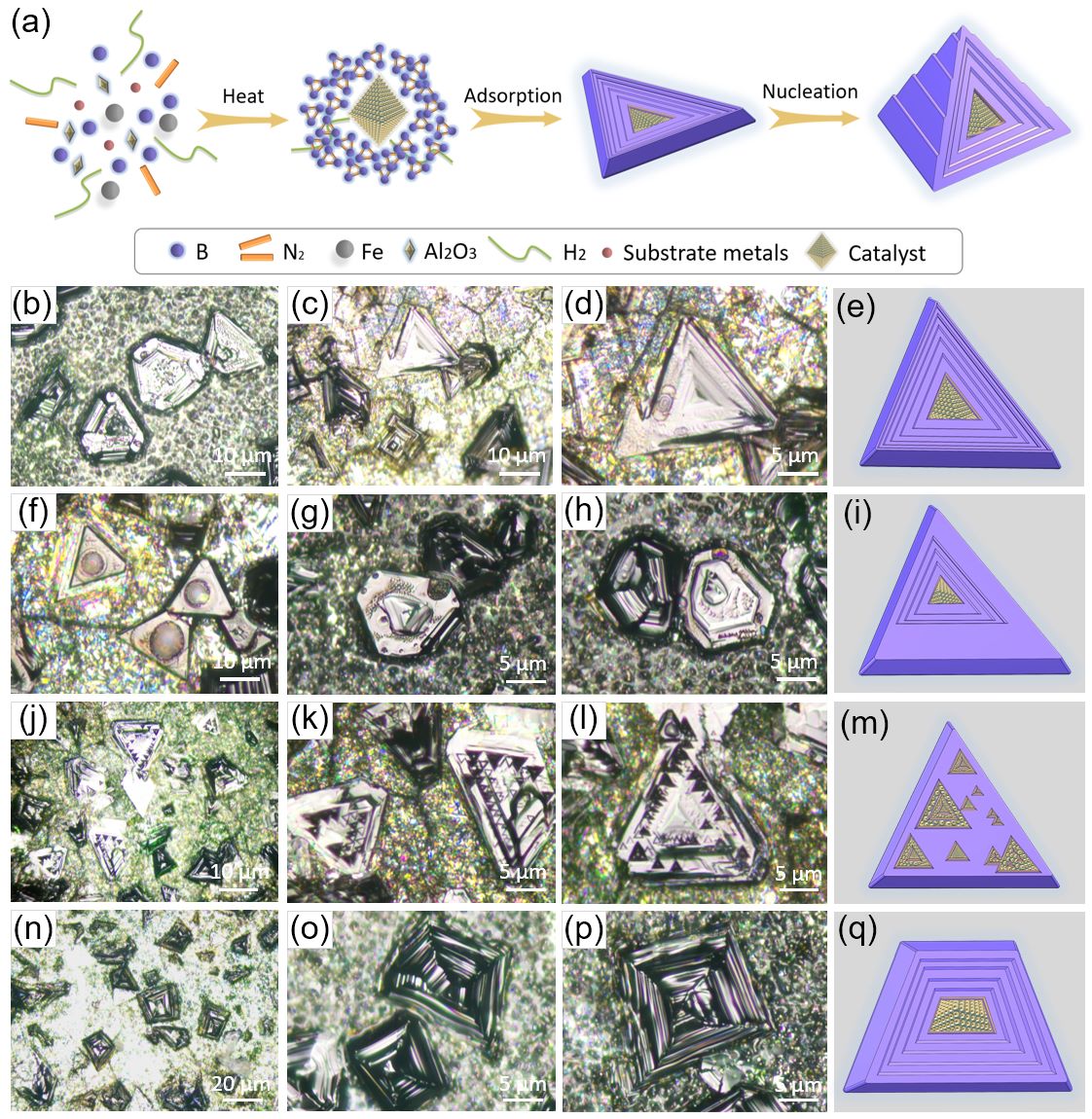


**Figure S6.** Images and schematic diagram of the nucleation stage of BNNTs under optical microscopy. a) Schematic diagram of the formation principle of BNNTs nuclei at the initial stage. The B and N sources are adsorbed and decomposed by the catalyst, forming BN clusters that surround the catalyst surface, creating regular-shaped nuclei. The nucleation points are formed by BN surrounding the catalyst. b-d) Triangular nuclei with the initial nucleation points at the center and their corresponding schematic diagrams. From the center, a layer-by-layer aggregation of triangular nuclear layers is visible, which is more clearly shown in the schematic diagram (e). f-i) Triangular nuclei with the initial nucleation points at the edges and their corresponding schematic diagrams. The nucleation points are at the edges of the triangle, exhibiting triangular layered or liquefied (or semi-liquefied) circular Fe nucleation points. j-m) Triangular nuclei with multiple nucleation points and their corresponding schematic diagrams. The surface of the triangular nuclei has multiple nucleation points in the shape of triangles, mainly concentrated at the center or arranged along the edges. n-q) Quadrilateral nuclei with the initial nucleation points at the center and their corresponding schematic diagrams. From the center, nucleation points are formed in layered quadrilateral shapes.

The BNNTs nuclei have different regular shapes, which are related to the surface structure of the metal substrate, the distribution of the catalyst, and the interfacial energy (the interface energy between the catalyst, source material, and metal substrate).^[2, 3]^ At the same time, the nucleation and growth of BNNTs are also influenced by the crystal structure of boron nitride itself. Hexagonal boron nitride (h-BN) has a hexagonal crystal structure similar to graphene, and the resulting layers have high symmetry.^[4, 5]^ Figure S6b-d show the triangular nuclei at the initial stage of BNNTs formation. It can be observed that the surface of the nuclei has layered triangular nucleation points, which is caused by the aggregation of the catalyst at the center of the nucleus, with BN allotropes and source material surrounding it. Figure S6e is a schematic diagram of the triangular nuclei morphology, providing a more intuitive understanding of the nucleus structure. Figure S6f-i show triangular nuclei with the initial nucleation points at the edges and their corresponding schematic diagrams. The nucleation points are at the edges of the triangle, showing triangular layered or liquefied (or semi-liquefied) circular Fe nucleation points. Figure S6j-m also show triangular nuclei at the initial stage of BNNTs formation, but the nucleation points on the surface are not completely concentrated at the center but are distributed across the surface. It can be observed from the figure that, in addition to the layered triangular shape in the center, triangular nucleation points are also arranged along the edges. This is due to the fact that the catalyst has not completely aggregated at the center of the nucleus but is instead distributed across the surface. Figure S6n-q show quadrilateral nuclei at the initial stage of BNNTs formation. Similar to Figure S6b-d, the catalyst aggregates at the center of the nucleus, adsorbing BN allotropes and source material to form a layered distribution.

In the early stage of nucleation, different crystal planes and orientations may form different geometric shapes. Different symmetries lead to different nucleation morphologies, and the hexagonal crystal system can generate triangular and quadrilateral core structures, which may evolve into triangular and quadrangular pyramids during further growth. A large number of triangular and quadrangular pyramidal nuclei were indeed found on the surface of the metal substrate where BNNTs were generated (as shown in Figure S7). The surface state of the metal substrate (such as flatness, chemical composition, and oxide layer) influences the nucleation morphology. External disturbances (such as airflow, vibrations, etc.) may affect the symmetry and uniformity of the nucleation morphology.^[6]^ The shape and size of nano-Al_2_O_3_ and Fe particles also directly influence the nucleation morphology. Particles with different shapes of catalysts can induce different nucleation shapes on their surface.^[7]^ Uneven distribution of catalyst particles can lead to local differences during the nucleation process, resulting in different geometric shapes and surface conditions. Figure S7 shows the initial BNNTs nuclei in the form of triangular and quadrangular pyramids. Based on our observations, the triangular and quadrangular pyramidal initial BNNTs nuclei evolved from triangular and quadrilateral nucleation structures. Additionally, identical or different types of nuclei can overcome interfacial barriers and fuse with each other.


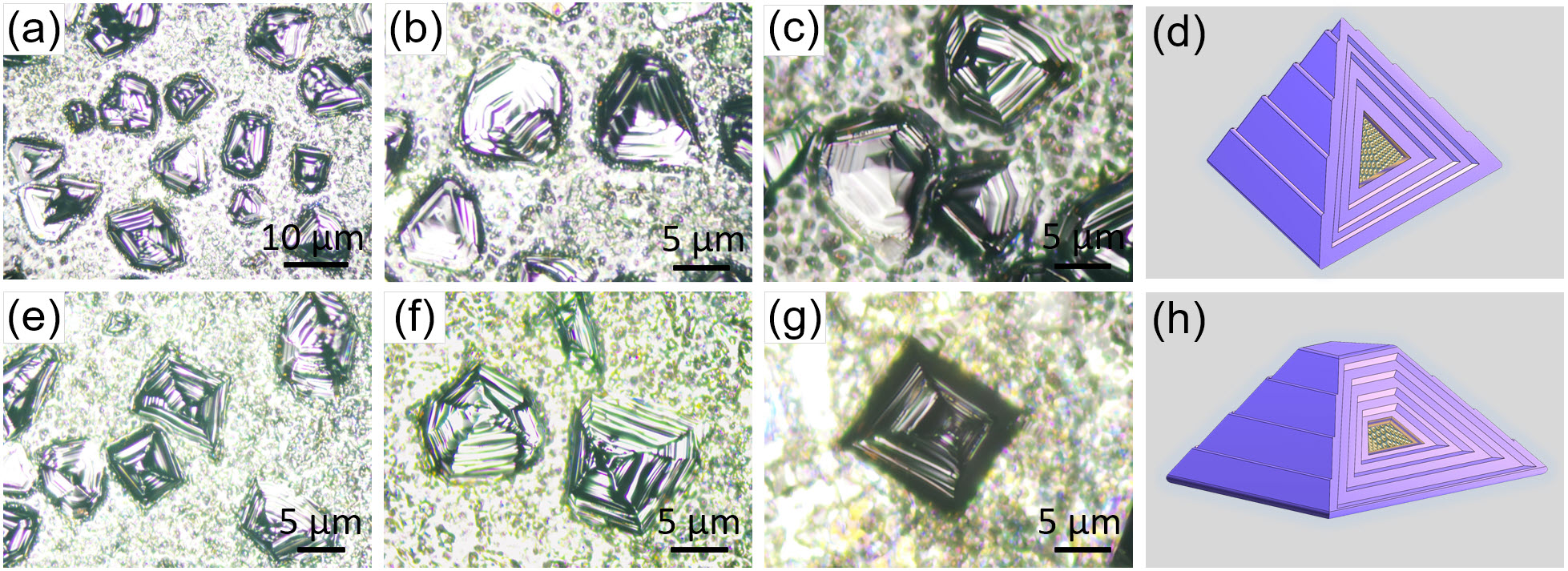


**Figure S7.** Images and schematic diagram of the initial nucleation of BNNTs in triangular and tetrahedral shapes under optical microscopy. a-c) Triangular pyramid-shaped BNNT nuclei exhibiting layer-by-layer stacking from the center toward the edges. d) Schematic illustration of the triangular pyramid morphology. e-h) Quadrangular pyramid-shaped BNNT nuclei and corresponding schematic representation.

To further reveal the structural characteristics of the nuclei, we used SEM to observe the initial BNNTs nuclei of different morphologies. Figure S8 shows the SEM images of the initial BNNTs nuclei. Comparative analysis revealed that the nuclear morphologies are highly consistent with the surface structures captured by optical microscopy in Figure S6 and S7. Figure S8a-h show SEM images of nuclei with different morphologies, while Figure S8i-l illustrate the complete process from initial nuclei to the activation of nucleation sites on the nuclei surface. Specifically, Figure S8i shows the morphology of the nuclei at the initial stage. Figure S8j, k depict the process where the nuclei break through boundaries and merge to form clusters. Figure S8l indicates that the nucleation sites on the nuclei surface are activated, leading to the generation of BNNTs, which gradually cover the nuclei and clusters, marking the initiation of the third phase of BNNTs formation (i.e., the interweaving of nascent BNNTs forming a hotbed for further growth).


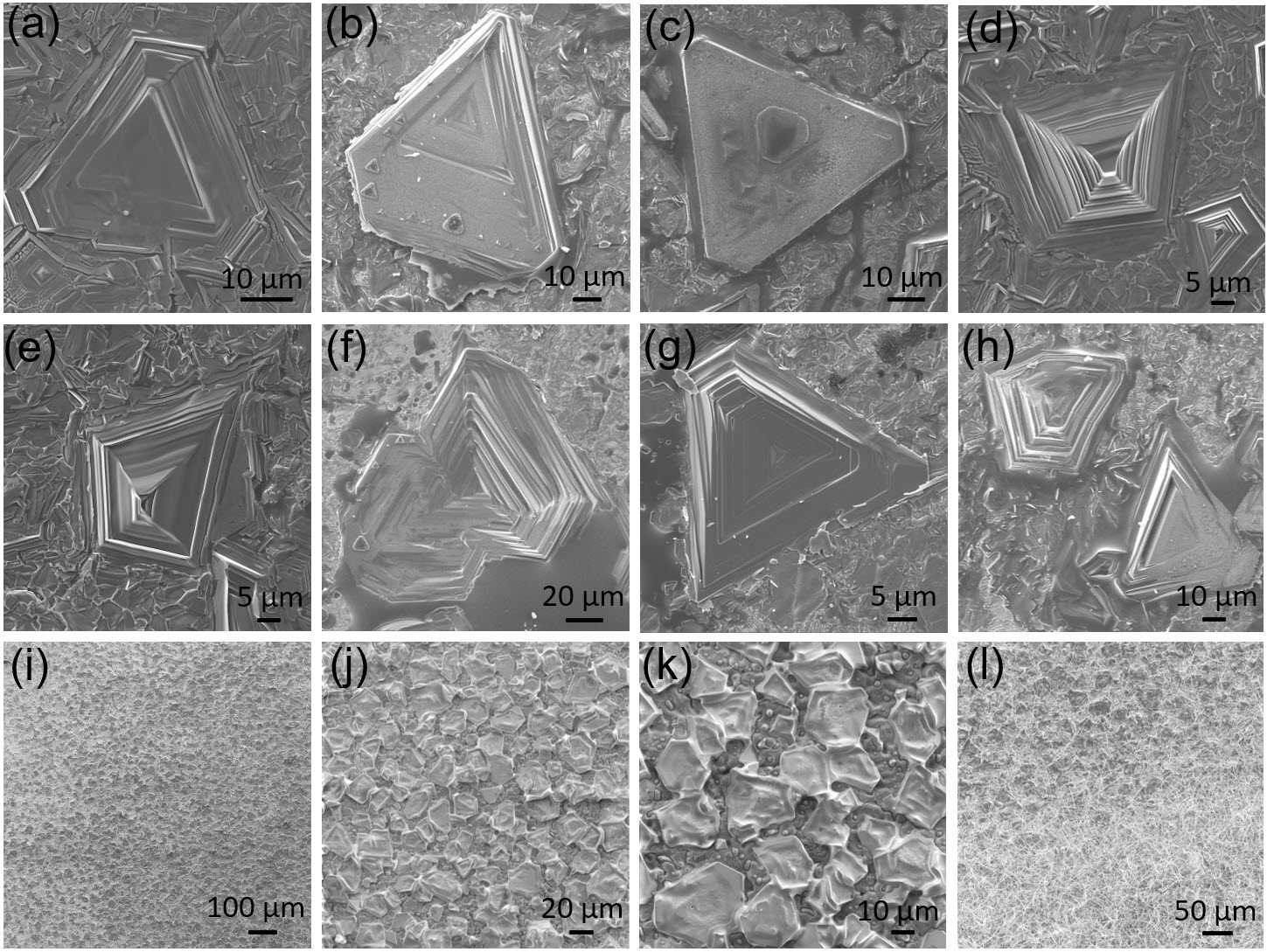


**Figure S8.** SEM images of initial BNNTs nuclei. a) Triangular nucleus with catalysts aggregated at the center. b) Triangular nucleus with catalysts aggregated at the edges. c) Fe catalyst liquefied (or semi-liquefied) into a round nucleation point and Al_2_O_3_ distributed at the edges of the triangular nucleus. d) Quadrilateral nucleus with catalysts aggregated at the center. e) Evolved into a quadrangular pyramidal nucleus. f) Two triangular nuclei breaking through boundary limits to merge into a cluster. g, h) Evolved into triangular pyramidal nuclei. i-l) SEM images showing the excitation of nucleation on the nucleus surface from initial BNNTs nuclei. i) Initial nuclei. j, k) Nuclei breaking through boundaries to merge into clusters. l) Nucleation points on the nuclei surface being excited to form BNNTs.

The morphological characteristics of BNNT nuclei are significantly correlated with the size and distribution of the catalyst. Furthermore, the morphology of BNNTs is strongly regulated by catalyst size and distribution. Smaller catalyst particles promote the formation of fine-diameter, high-density BNNT clusters by increasing the density of nucleation sites, whereas larger particles tend to generate coarse-diameter, low-density structures.^[8]^ A uniform catalyst distribution ensures the regular alignment and morphological uniformity of nanotubes, while non-uniform distribution can lead to local aggregation or branching.^[9, 10]^ This synergistic effect of size and distribution also influences the crystallinity and defect density of BNNTs. Therefore, precise control of catalyst parameters is critical for the controlled synthesis of BNNT morphology.

**S4. Growth Clusters of BNNTs**

The second stage of BNNT growth is the fusion of the nuclei and the gradual growth of BNNTs. During this stage, the nucleation sites on the surface of the nuclei are activated, initiating the formation of BNNTs. This process is highly complex, involving the role of the catalyst, surface diffusion and accumulation, gas-phase and surface reactions, growth kinetics, as well as crystallization and morphology control. The active sites provided by the catalyst, together with the appropriate reaction conditions, drive the continuous growth and morphological evolution of BNNTs. By precisely controlling these key factors, effective control over the BNNT growth process can be achieved, enabling the fabrication of BNNTs with specific structures and properties.

Catalysts play a crucial role in the growth of BNNTs. For example, catalysts such as Fe, Ni, and Co can react with boron and nitrogen under high-temperature conditions to form transition-state metal-boron alloys. These alloys not only provide active sites but also further promote the continuous growth of BNNTs.^[11]^ During the high-temperature reaction, the morphology of the catalyst particles may undergo significant changes, such as transitioning to a liquid or semi-liquid form, which creates a microenvironment conducive to BNNT growth. This environment facilitates the nucleation and expansion of BN allotropes on the catalyst surface, further promoting the formation of BNNTs. Figure S9 illustrates the second stage of BNNT growth: the gradual fusion of the nuclei to form clusters, followed by the generation of BNNTs upon the activation of nucleation sites on the surface (Figure S9a-c). As the reaction proceeds, the resulting BNNTs gradually cover the surface of the nuclei. As shown in Figure S9d-f, after the nucleation sites on the surface of the nuclei are activated, BNNTs gradually cover the nuclei, marking the transition of BNNT growth into the third stage, the so-called BNNTs hotbed phase.


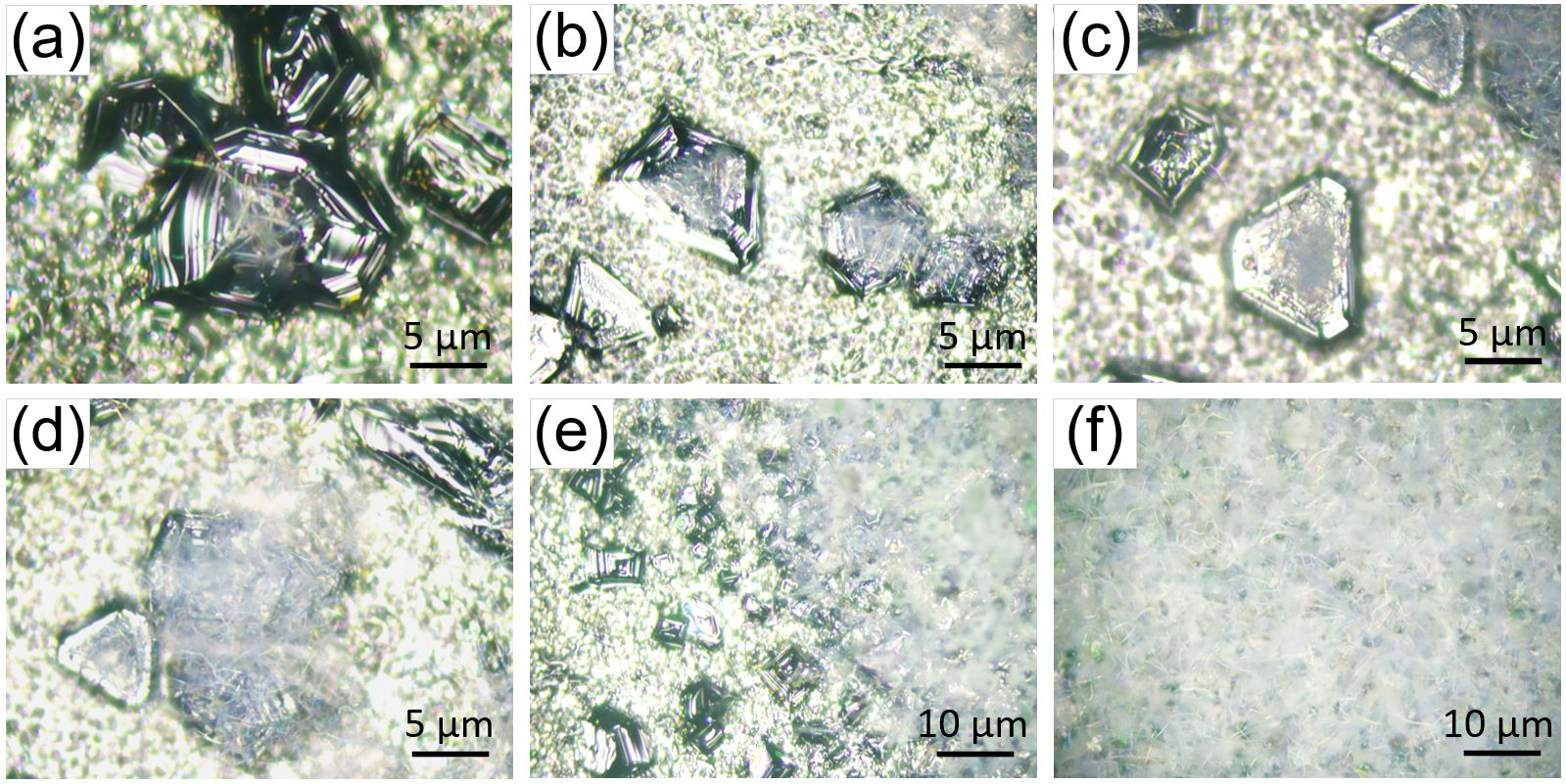


**Figure S9.** Optical microscope image of the clusters and nucleation sites triggering growth during the second stage of BNNTs growth. a) Fusion occurs between nuclei, and surface nucleation points are excited to form BNNTs. b) Surface nucleation points of quadrilateral nuclei are excited to form BNNTs. c) Surface nucleation points of triangular nuclei are excited to form BNNTs. d) The formed BNNTs gradually cover the nuclei. e) Boundary between BNNTs-covered nuclei and uncovered nuclei after large-scale excitation. f) Nuclei are completely covered by BNNTs formed on the surface.

Furthermore, our study shows that the pre-treatment of metal substrates significantly increases the number of BNNTs nuclei and the density of nucleation sites on their surface. This improvement is primarily attributed to the enhancement of the smoothness and uniformity of the metal substrate surface, which increases surface energy and active sites. In addition, the pre-treatment removes surface contaminants and oxide layers, allowing the catalyst to better adhere to and evenly distribute on the metal surface. These factors work together to ensure that electrochemically polished metal substrates exhibit superior performance during the nucleation and growth of BNNTs, thereby significantly enhancing nucleation point density and growth quality.

Figure S10 shows the energy dispersive X-ray spectroscopy (EDS) spectrum of the BNNTs growth clusters. As can be seen from the figure, the surface is primarily populated by Al_2_O_3_-based catalysts. As the core catalyst, Al_2_O_3_ not only provides stable support but also, through its synergistic effect with other metal catalysts, effectively lowers the energy barrier during nucleation and growth, thereby promoting the formation of BNNTs. Additionally, the Al_2_O_3_ surface exhibits low surface energy and abundant active sites, which significantly enhance its adsorption and reactivity with B and N, providing favorable conditions for the nucleation of BNNTs.


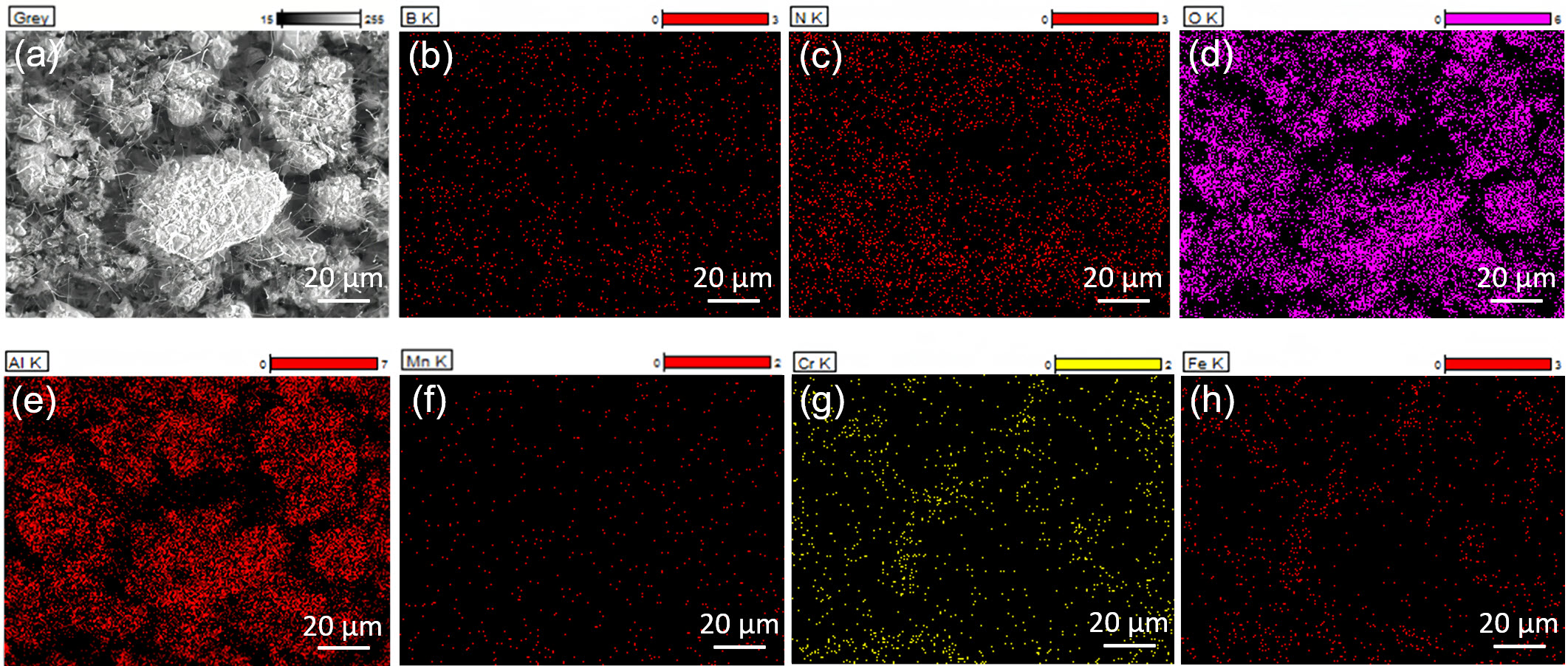


**Figure S10.** EDS spectrum of BNNTs growth clusters. a) SEM image of the scanned region. b) B element. c) N element. d) O element. e) Al element. f) Mn element. g) Cr element. h) Fe element.

**S5. Patterning of BNNTs and Metal Mesh Substrate Preparation**

To validate the controllability and scalability of this BNNT synthesis method, we utilized a brush-painting approach to achieve patterned growth of BNNTs on metal substrates and successfully grew BNNTs on metal meshes. Figure S11 illustrates the "HIT" pattern of BNNT growth and its edges. The white product in the upper right corner of Figure S11a represents the patterned BNNTs, and the orange box highlights an enlarged view of the boundary between the boronized and non-boronized regions. Figure S11b shows an enlarged edge image, revealing a well-defined boundary, indicating that this method offers strong controllability and diverse application potential in BNNT synthesis.


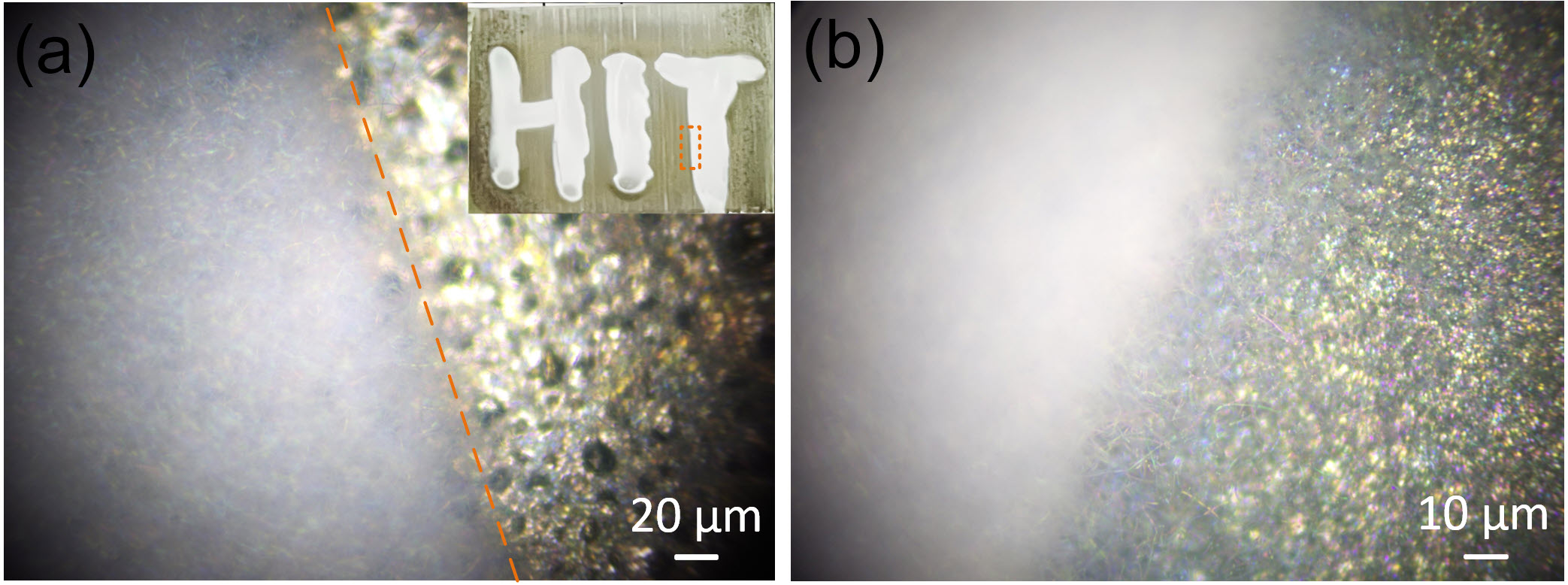


**Figure S11.** Optical microscope images of patterned BNNTs and their patterned edges.

In addition, we successfully fabricated BNNTs on pretreated stainless steel metal mesh. Figure S12a, b show images of BNNTs grown on stainless steel metal mesh, with the mesh covered by white BNNTs. Figure S12c, d display BNNTs grown at different positions on the metal mesh. It can be observed that due to the porous nature and excellent thermal conductivity of the stainless-steel mesh, the contact between the source gas and B source is more thorough, and heat distribution is more uniform. This increases the efficiency of BN allotropes eneration, resulting in more uniform BNNT growth with lower impurity levels (such as unreacted B and unformed h-BN). In addition to more efficient B source reactions, the synthesized BNNTs have more uniform lengths and diameters, likely due to the porous nature and excellent thermal conductivity of the stainless-steel mesh. The porous characteristics help the gas source penetrate the surface of the stainless-steel mesh, increasing the formation rate of BN allotropes. The good thermal conductivity of the mesh helps evenly distribute heat during growth, preventing localized overheating and thus promoting uniform BNNT growth.


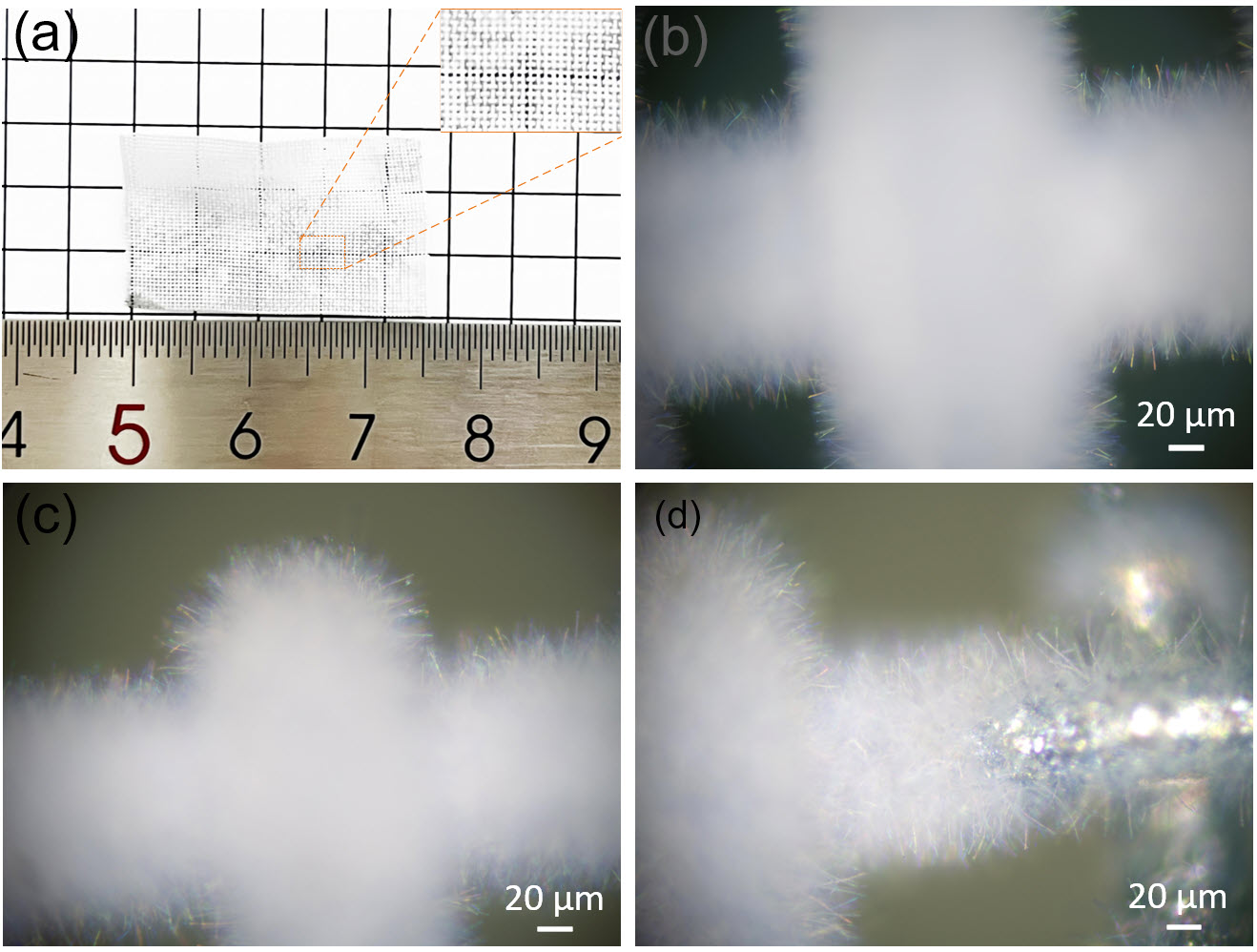


**Figure S12.** Macroscopic and optical microscope images of BNNTs grown on a stainless-steel mesh substrate. a) BNNTs grown on stainless steel mesh. b) BNNTs grown at the junction of the stainless-steel mesh. c) BNNTs grown at the edge of the stainless-steel mesh. d) Boundary between the region with BNNTs grown on the stainless-steel mesh and the region without BNNT growth.

**S6. Transfer, Length, and Impurity Distribution of BNNTs**

To promote the practical application of BNNTs, we used thermal release tape (TRT) technology to peel and transfer them layer by layer onto Si/SiO_2_ substrates for further parameter studies. In addition, BNNTs are required to have extremely high purity in practical applications. Therefore, unreacted B, BN allotropes, catalysts (such as nano-Al_2_O_3_ and Fe), as well as residual Cr and Ni from the metal substrate, are considered impurities.


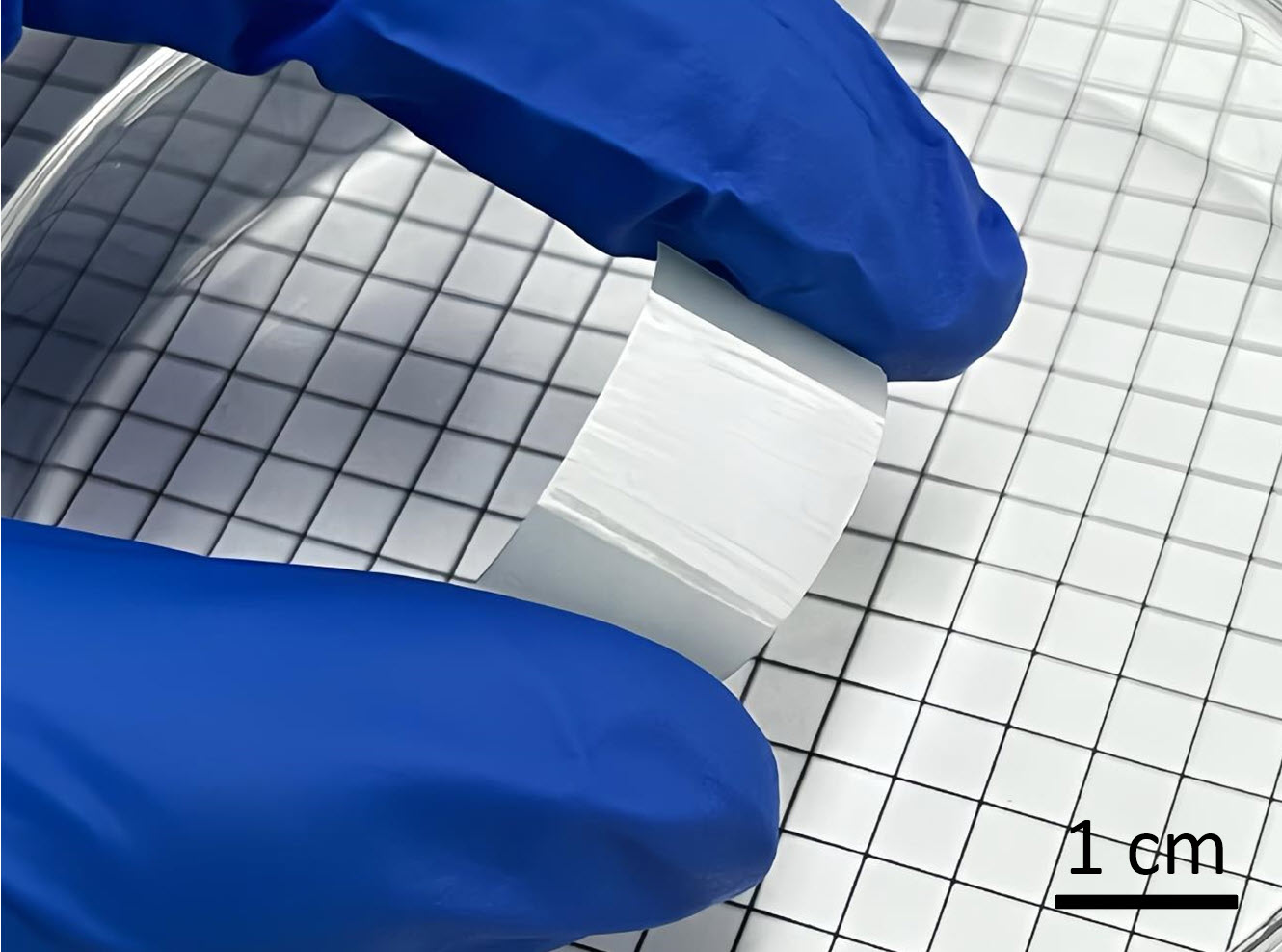


**Figure S13.** BNNTs on TRT.

To facilitate the observation of the length parameters of BNNTs, we bonded thermal release tape to a Si/SiO_2_ substrate and used heating to release and transfer the BNNTs onto the Si/SiO_2_. As shown in Figure S13, the BNNTs are adhered to the surface of the thermal release tape and transferred to the Si/SiO_2_ substrate through heating, as shown in Figure S14. From the figures, it can be seen that the dense BNNTs prepared on the metal substrate surface exhibit differences in tube length at different levels, with impurities primarily concentrated inside the hotbed. The specific length statistics are shown in Figure S15. The average length of top-layer BNNTs is L_avg_ = 36 μm, the average length of BNNTs on the metal substrate surface after the fourth peel-and-transfer process is L_avg_ = 47 μm, the average length of BNNTs on the hotbed surface is L_avg_ = 65 μm, and the average length of BNNTs inside the hotbed is L_avg_ = 28 μm. We found that the BNNTs on the surface of the hotbed were the longest, while the BNNTs inside the hotbed were shorter. The growth process of BNNTs from nucleation to the g hotbed is a preparatory phase for their growth. During this phase, the catalyst and B source are highly concentrated. Due to unstable reaction conditions, immature catalysts, and the negative effects of reaction kinetics, the BNNTs are shorter and contain more impurities. As the reaction conditions stabilize, the catalyst matures, and the source supply becomes sufficient, BNNTs can grow in a more stable and ordered manner, leading to increased length and reduced impurities.

Figure S16 systematically characterizes the length distribution, diameter uniformity, and wall thickness features of BNNTs. In Figure S16a, the average length distribution of BNNTs from the top layer to the substrate indicates that the BNNTs in the top layer are shorter than those on the surface of the substrate. We attribute this phenomenon to the influence of the upper-layer BNNTs at later growth stages, which may hinder gas flow from reaching the substrate interior, leading to insufficient precursor supply. The TEM image in Figure S16b illustrates the uniform tubular structure of BNNTs, with a stable diameter of approximately 100 nm, confirming the effective control of radial dimensions during synthesis. Figure S16c, d present a quantitative analysis of BNNT wall thickness through magnified TEM images. The measured wall thickness at different positions is 12.01 nm at the bamboo-like segment's front end and 38.43 nm at the rear end, indicating a gradient in wall thickness within the same nanotube.


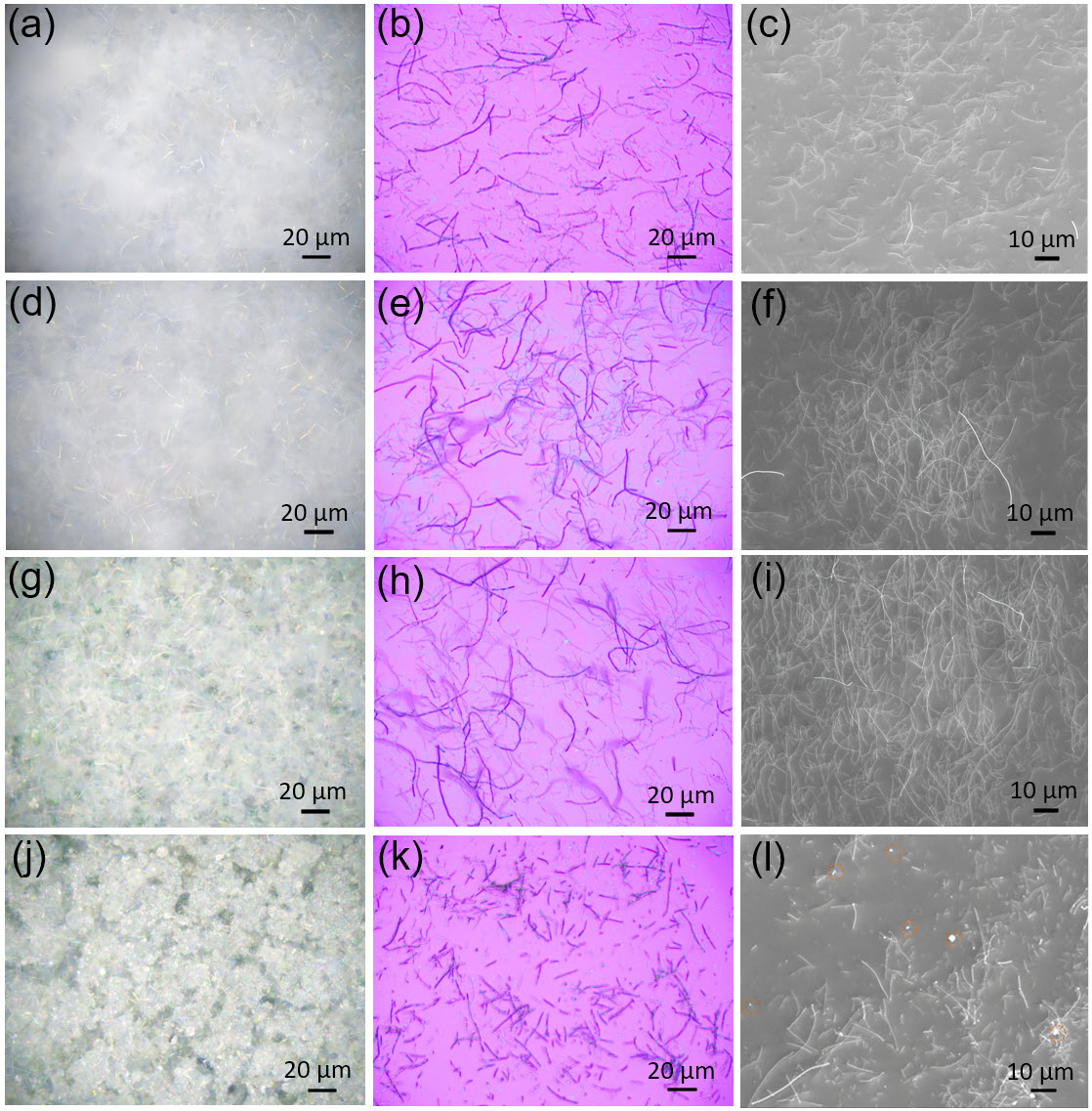


**Figure S14.** Shows the dense BNNTs on the metal substrate peeled off with TRT. a) Optical microscope image of top layer BNNTs (first peel and transfer). b, c) Optical microscope and SEM images of top layer BNNTs transferred to the SiO_2_ surface after the first peel with TRT. d) Optical microscope image of BNNTs on the metal substrate surface after the fourth peel and transfer. e, f) Optical microscope and SEM images of BNNTs on the metal substrate surface transferred to the SiO_2_ surface after the fourth peel with TRT. g) Optical microscope image of BNNTs on the surface of the bedding layer. h, i) Optical microscope and SEM images of BNNTs on the surface of the bedding layer transferred to the SiO_2_ surface with TRT. j) Optical microscope image of BNNTs inside the bedding layer. k, l) Optical microscope and SEM images of BNNTs inside the bedding layer transferred to the SiO_2_ surface with TRT. Impurities are indicated by the orange circles.


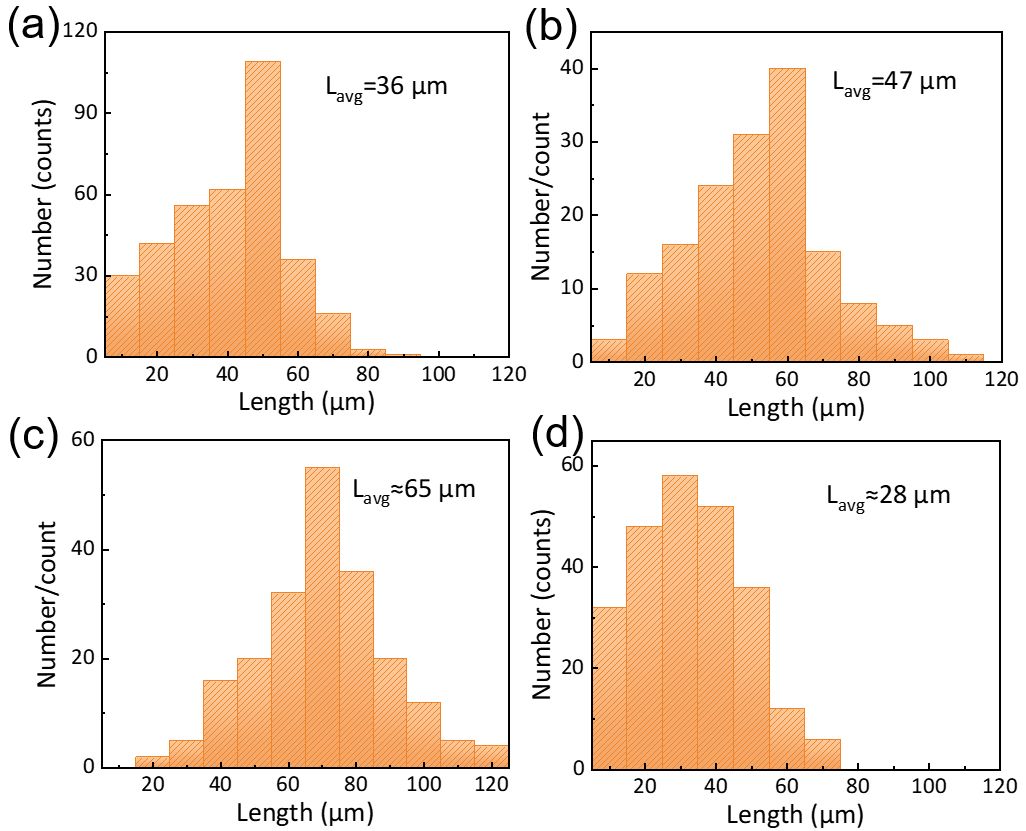


**Figure S15.** Length distribution of dense BNNTs on the metal substrate. a) Length distribution of top layer BNNTs. b) Length distribution of BNNTs on the metal substrate surface after the fourth peel and transfer. c) Length distribution of BNNTs on the surface of the bedding layer. d) Length distribution of BNNTs inside the bedding layer.


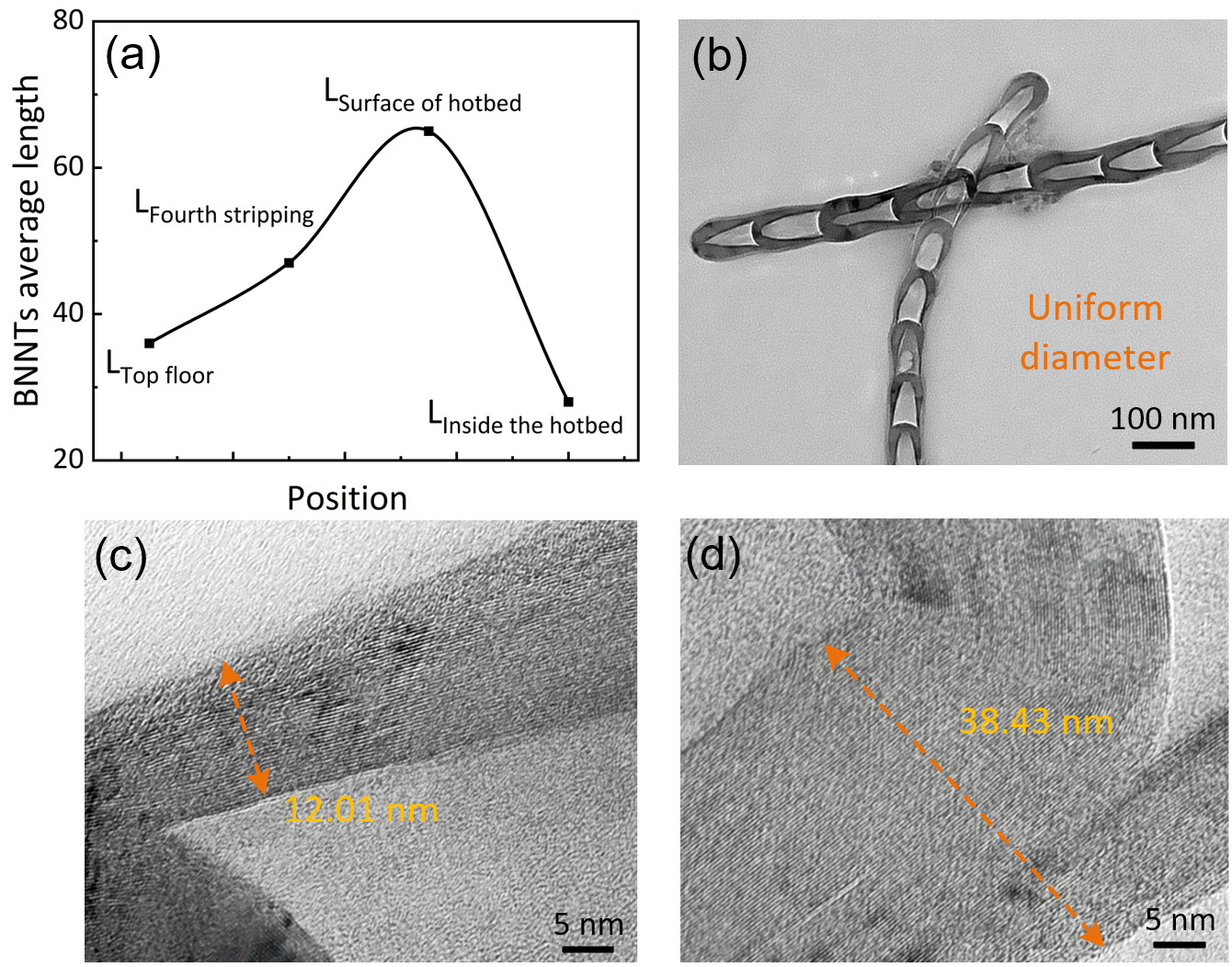


**Figure S16.** Length distribution and diameter parameters of BNNTs. a) Average length distribution of BNNTs at different positions. b) TEM image showing the uniform structure and diameter of BNNTs. c, d) Wall thickness of BNNTs at different positions.

**S7. Purification of BNNTs**

Purifying BNNTs is crucial for their applications, as high-purity BNNTs exhibit better performance and reliability,^[12, 13]^ and are of significant importance in advanced materials and devices. Different preparation methods result in varying types and amounts of impurities in BNNTs. The BNNTs produced by this method mainly contain unreacted B, catalysts, and h-BN that has not formed BNNTs. To more intuitively observe the impurities, we also achieved the vertical growth of BNNTs (as shown in Figure S17a). Thanks to the vertical growth method, we can observe from top to bottom that the tips of the BNNTs mainly contain the catalyst as an impurity.^[14]^ The orange circles or curved regions in Figure S17b and c are populated with numerous black dot-like structures, further indicating the distribution of impurities.


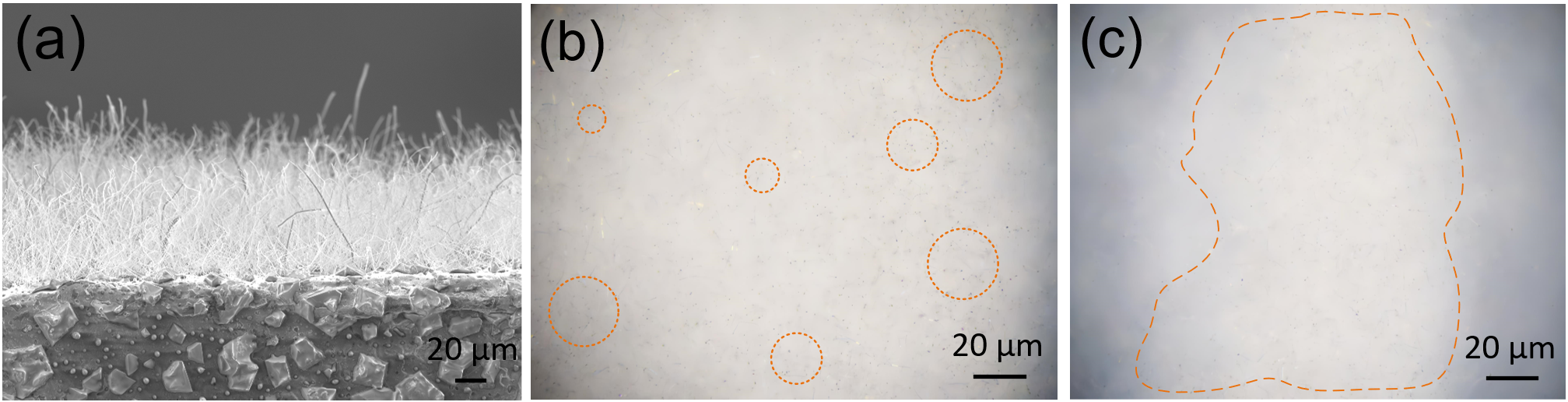


**Figure S17.** Vertical growth of BNNTs and impurities at the tips. a) SEM image of the vertical growth of BNNTs. b, c) Optical microscope image of the impurities at the tips of vertical BNNTs (area enclosed by the orange curve).

Unreacted B and h-BN mainly accumulate inside the hotbed where BNNTs grow extensively, and catalyst impurities (such as Al_2_O_3_, Fe, and Cr and Ni on the metal substrate surface) are also present. To remove boron impurities and metal particles (including catalysts and ball milling debris) associated with BNNTs synthesis, we employed a concentrated acid reflux method, commonly known as "leaching".^[15]^ In this process, a 3M hydrochloric acid (HCl) solution was used, and the duration and temperature of the oxidation reaction were controlled to prevent damage to the BNNTs, thereby effectively removing the metal particles generated during the preparation process.^[16]^ The main reactions of this process are as follows:

Al_2_O_3_ reacts with HCl to form aluminum chloride and water:

$Al_{2} O_{3} +6HCl\to2AlCl_{3} +3H_{2} O$ (5)

*Fe* reacts with HCl to form ferrous chloride and hydrogen gas:

$Fe+2HCl\to FeCl_{2 }+H_{2}$ (6)

In the air, *Fe^2+^* can be further oxidized to *Fe^3+^*:

$4FeCl_{2} +O_{2} +4HCl\to4FeCl_{3 }+2H_{2} O$ (7)

*Cr* reacts with HCl to form CrCl_2_ and *H_2_*:

$Cr+2HCl\to CrCl_{2} +H_{2}$ (8)

*Cr^2+^* can also be further oxidized to *Cr^3+^*:

$2CrCl_{2} +2HCl+H_{2} O_{2} \to2CrCl_{3 }+2H_{2} O$ (9)

*Ni* reacts with HCl to produce nickel chloride and H_2_:

$Ni+2HCl\to NiCl_{2} +H_{2}$ (10)

As shown in Figure S18a, during the leaching of the prepared BNNTs in HCl solution, the left side of the HCl solution turned green, indicating that metals such as Fe, Cr, and Ni were dissolved. Figure S18b shows the unpurified BNNTs, where impurities (highlighted by red circles) are clearly visible. BNNTs and h-BN are chemically stable in HCl and do not undergo any chemical reactions; however, catalyst impurities react significantly in HCl, producing products such as FeCl_2_, FeCl_3_, CrCl_3_, AlCl_3_, and NiCl_2_. These products dissolve in hot water and dissociate into the corresponding metal ions and chloride ions.^[16-18]^ Due to the hydrolysis of metal ions, some ions will further form hydrates or hydrolysis products. Therefore, to remove catalyst impurities, repeated washing with hot water is required.


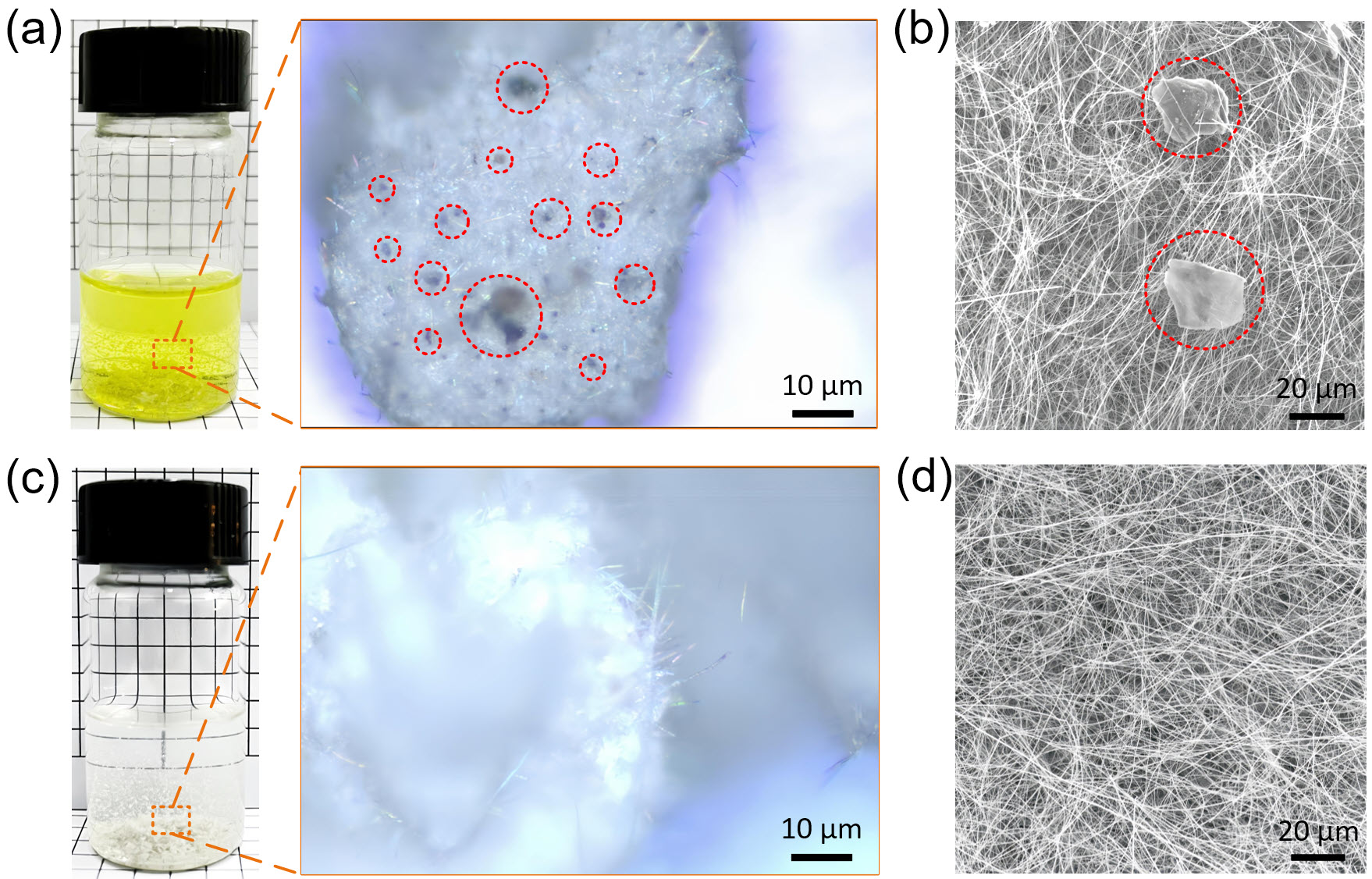


**Figure S18.** Purification of BNNTs. a) Optical microscope image of BNNTs cleaned in HCl. b) Purified BNNTs.

To thoroughly remove the impurities in BNNTs, we performed dispersion filtration treatment on the BNNTs that were washed with hot water. Sodium deoxycholate (DOC) effectively reduces the van der Waals forces between BNNTs, preventing aggregation and ensuring uniform dispersion of BNNTs in the solution.^[19]^ Through filtration, the BNNTs are retained on the filter membrane, while dissolved impurities and other small particles (such as residual metal catalysts, unreacted precursors, and other minute impurities) pass through the filter membrane, achieving separation. Figure S18c shows the mixed solution of purified BNNTs and ethanol, while Figure S18d displays the SEM image of the purified BNNTs, showing a smooth and uniform surface with no visible impurities.

In addition, before the acid leaching, we first removed the unreacted amorphous B and nitrides by high-temperature treatment. We performed thermogravimetric analysis (TGA) on the BNNTs before purification (BP-BNNTs), after purification (AP-BNNTs), h-BN, B_2_O_3_, and B to determine the oxidation and decomposition temperatures of various impurities in the BNNTs samples, and to assess the purity and thermal stability of the BNNTs before and AP-BNNTs.^[13]^ Figure S19a shows the TGA curves of several samples (10°C/min to 1200°C). It can be observed that B shows a noticeable mass increase after 300°C, indicating oxidation. B_2_O_3_ and BP-BNNTs also undergo slight decomposition or oxidation at this point (after 300°C). This indicates that the small amounts of B_2_O_3_ and unreacted B in BP-BNNTs underwent decomposition or oxidation. After 600°C, B_2_O_3_ becomes stable, indicating that B_2_O_3_ has completed all possible oxidation, decomposition, or dehydration processes, and no further physical or chemical processes that would cause mass changes occur. BP-BNNTs and h-BN show a noticeable mass increase after 900°C, indicating that the h-BN in BP-BNNTs and h-BN underwent oxidation. The mass of AP-BNNTs only shows significant changes near 1000°C, indicating that AP-BNNTs exhibit higher thermal stability, with less mass loss at low and intermediate temperatures, suggesting that impurities have been effectively removed. This further confirms the main components of the BNNTs and their purity.


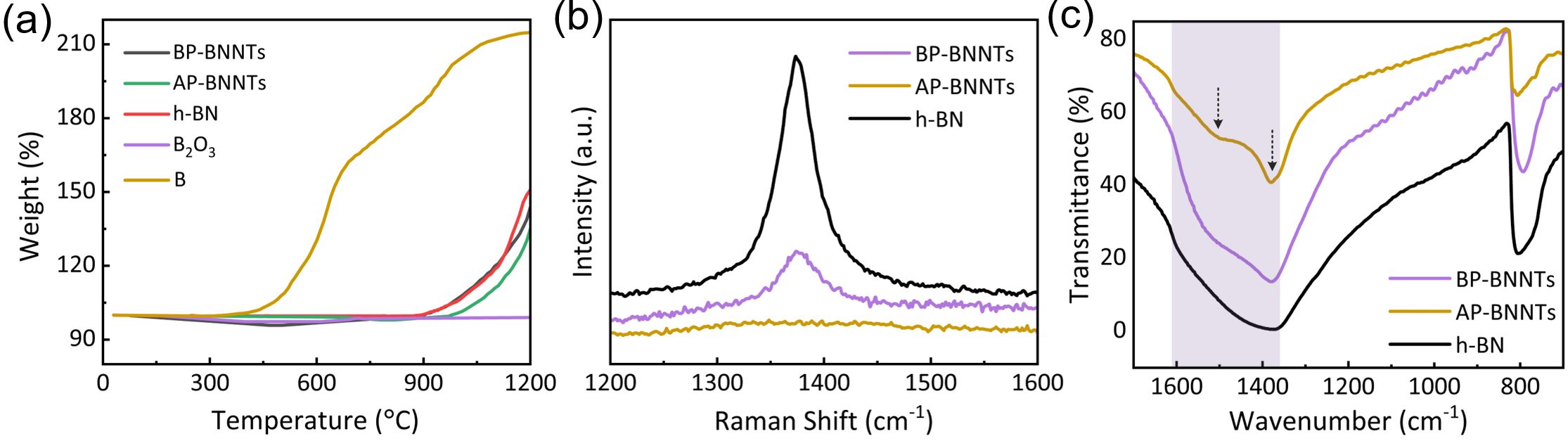


**Figure S19.** Characterization of BNNTs before and AP-BNNTs. a) Comparison of TGA of BNNTs before and AP-BNNTs and different impurities. b) Non-resonant Raman spectra of BNNTs before and AP-BNNTs and h-BN. c) Comparison of FTIR spectra of BNNTs before and AP-BNNTs and h-BN.

To further evaluate the purification quality of BNNTs, we performed non-resonant Raman spectroscopy on the samples, distinct from the resonant Raman spectroscopy presented in Figure 1c. Due to the bandgap difference between BNNTs (5.5 eV, corresponding to a photon wavenumber of 44444.44 cm^-1^) and h-BN (5.9 eV, corresponding to 47619.05 cm^-1^), under resonant conditions near the BNNT bandgap (resonant Raman spectroscopy at 45662.10 cm^-1^), electronic transitions are resonantly excited, allowing the observation of characteristic peaks. However, under non-resonant conditions (when the excitation wavelength does not match the bandgap), the electronic transitions in BNNTs are not excited, resulting in an extremely weak or undetectable Raman signal. Interestingly, although the excitation wavelength in non-resonant Raman spectroscopy (e.g., 19455.44 cm^-1^ or 15795.74 cm^-1^ ) does not match the bandgap of h-BN, its layered structure allows the E_2g_ vibrational mode (in-plane B-N bond vibration) to be detected via dipole moment variations under non-resonant conditions, resulting in a prominent peak at 1364.6 cm^-1^.^[13, 20]^ This phenomenon arises from the two-dimensional layered structure of h-BN, which supports active Raman modes that remain detectable even under non-resonant conditions. In contrast, the curvature and phonon mode variations induced by the tubular structure of BNNTs reduce the Raman activity of these vibrational modes. Some modes may shift or become Raman-inactive, leading to the absence of prominent peaks in BNNTs under non-resonant conditions, unlike h-BN. Therefore, non-resonant Raman spectroscopy can effectively distinguish h-BN from BNNTs, serving as a reliable method to assess the purification quality of BNNTs.

Figure S19b presents the non-resonant Raman spectra of BP-BNNTs, AP-BNNTs, and h-BN. A distinct Raman peak is observed at ~1365 cm^-1^ for BP-BNNTs, which is in perfect agreement with the characteristic peak of the E_2g_ in-plane vibrational mode of h-BN (literature value: 1364.6 cm^-1^),^[20]^ indicating the presence of h-BN impurities in the raw sample. In contrast, no characteristic peaks are detected in the non-resonant Raman spectrum of AP-BNNTs, demonstrating that the purification method effectively removed h-BN impurities.

To further verify the purification quality of BNNTs, we performed Fourier-transform infrared spectroscopy (FTIR) to detect residual impurities such as B_2_O_3_ and h-BN in the BNNTs.^[21]^ We also performed FTIR testing on BP-BNNTs, AP-BNNTs, and h-BN, as shown in Figure S19c. The h-BN impurities introduce additional absorption paths and complex vibrational modes, leading to peak broadening and an increase in peak intensity. During the purification process, these impurities were removed, resulting in a purer BNNT spectrum with clearer and more concentrated (narrower) characteristic peaks, which is consistent with the FTIR test results. Furthermore, compared to BP-BNNTs and h-BN, AP-BNNTs exhibited distinct double peaks within the RB band (black arrows in the figure), with lower peak intensity and narrower peak width, further confirming the efficiency of the purification process.

**S8. Directed Aligned Growth of BNNTs**

Aligned growth is an important milestone in the maturity of BNNTs synthesis methods. Although there are numerous studies on the aligned growth of CNTs, research on the aligned growth of BNNTs is still relatively scarce. Existing methods mainly use template-assisted approaches to achieve aligned growth of BNNTs.^[22, 23]^ In contrast, the method proposed in this paper is simple and straightforward, requiring no additional auxiliary materials or templates to achieve aligned BNNTs (ABNNTs). The detailed method is described in the Experimental section under BNNTs aligned growth.

Figure S20a shows a schematic of the ABNNTs at high temperature. After infiltrating the B-source mixture onto the treated metal substrate, the metal substrate is placed in a quartz boat (in the high-temperature zone of the tube furnace). To promote airflow along the grooves on the metal substrate surface, the metal substrate is suspended, and its alignment direction is consistent with the grooves. Figure S20b shows the grooves formed on the metal substrate surface after directional polishing treatment, where the B-source mixture can infiltrate along these grooves. At 1150°C, the nucleation sites on the BNNTs nuclei are influenced by the airflow, growing along the direction of the airflow, thereby achieving the growth of ABNNTs.


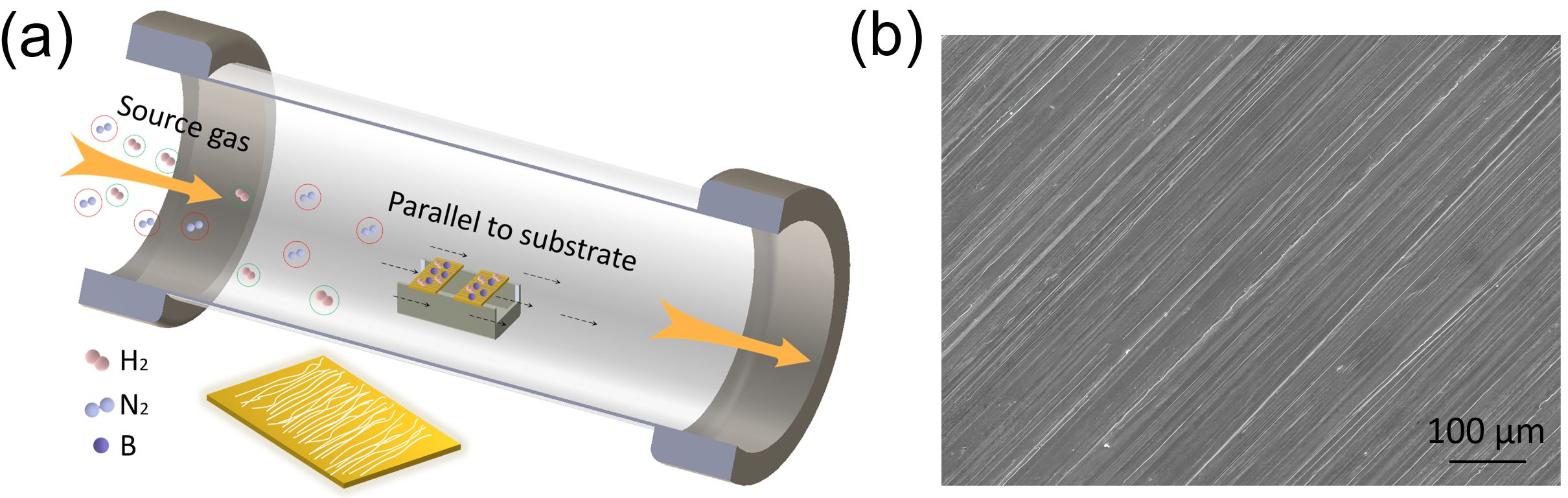


**Figure S20.** Growth of ABNNTs. a) Schematic diagram of aligned growth. b) SEM image of the metal substrate surface with neat grooves.

Figure S21 illustrates the morphological characterization and growth mechanism of directionally aligned BNNTs. Figure S21a presents a schematic of the directional growth mechanism, where BNNTs initially grow in arbitrary directions. Under the influence of airflow, they gradually exhibit aligned growth characteristics (nucleation clustering stage). Subsequently, airflow regulation facilitates the ABNNTs, ultimately leading to the formation of a large-area ordered structure (Large area aligned). Figure S21b-d present SEM images of ABNNTs at different scales (50 μm, 10 μm, and 50 nm) across various samples. The images demonstrate a high degree of orientation uniformity and structural consistency, confirming the reproducibility of alignment quality among different samples. The optical microscopy image in Figure S21e further validates the continuity and macroscopic order of the BNNT array at a mesoscale level. For comparison, Figure S21f, g show SEM images of BNNTs grown without directional control. The disordered morphology of these samples contrasts sharply with the ABNNTs, further demonstrating the effectiveness of the directional growth method.


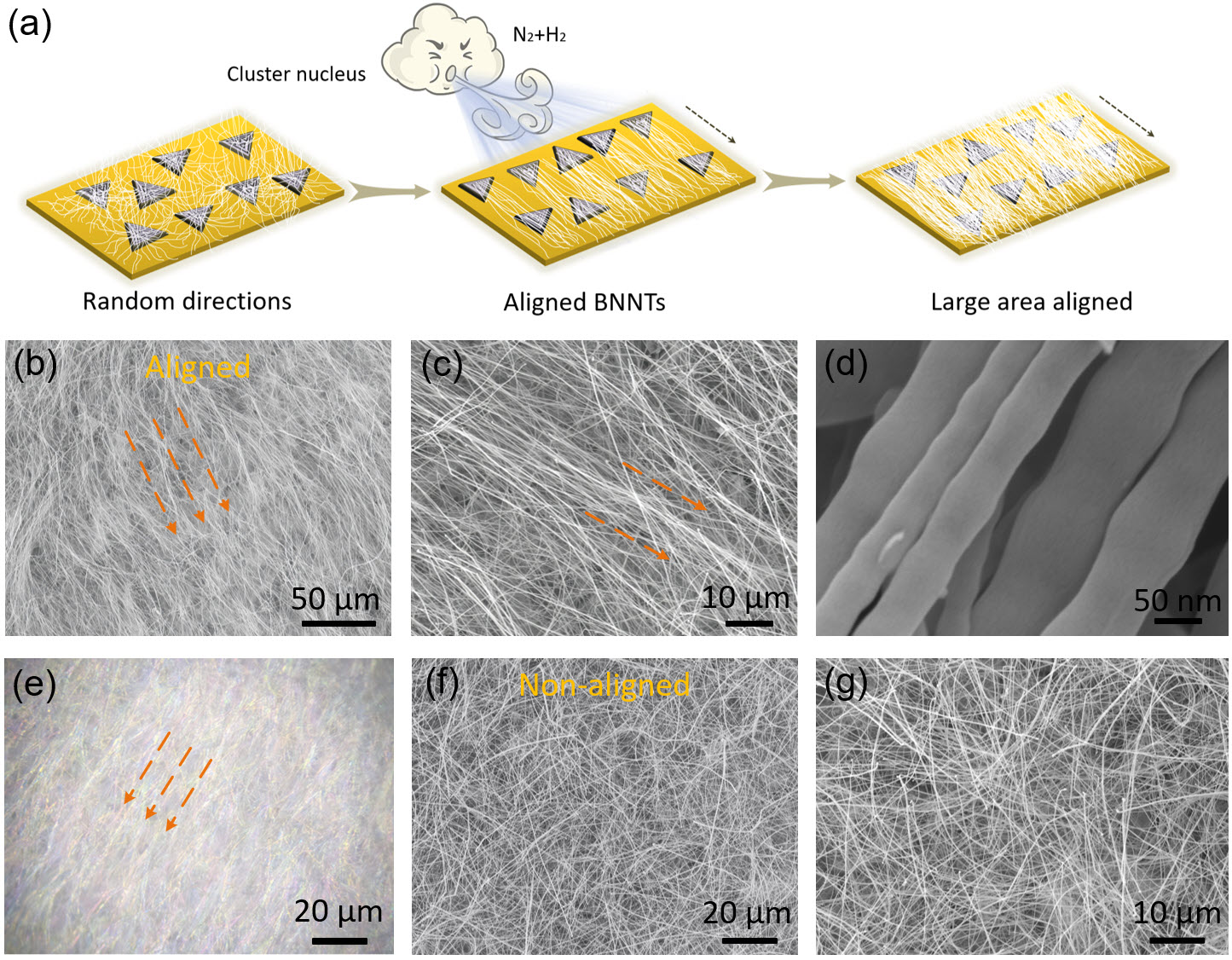


**Figure S21.** Images of the oriented growth of BNNTs. a) Schematic illustration of the oriented growth of BNNTs. b-d) SEM images of BNNTs grown with directional alignment. e) Optical microscope image of the aligned BNNTs. f, g) SEM images of BNNTs grown without alignment.

**S9. Optical Model of BNNTs in the Infrared Spectrum**

In the mid-infrared range, h-BN exhibits hyperbolic optical dispersion due to anisotropic$\varepsilon$, with associated two infrared-active phonon modes.^[24]^ There is a frequency band ($\omega_{TO}<\omega<\omega_{LO}$) between the transverse optical (TO) mode at$\omega_{TO}$(169.5meV) and the longitudinal optical (LO) mode at $\omega_{LO}$(200meV), where the in-plane dielectric constant of h-BN is $\varepsilon_{\parallel}<0$,^[25]^ and the out-of-plane dielectric constant is $\varepsilon_{\perp}>0$. This frequency range is known as the Reststrahlen band (RB) and is characterized by type-II hyperbolicity.

BNNTs are materials with a one-dimensional nanotube structure, which s can be obtained by rolling a sheet of h-BN into a tubular geometry. The out-of-plane component in planar h-BN is analogous to the radial one in a BNNT, $\varepsilon_{r}=\varepsilon_{\perp}$, while the in-plane component is analogous to the axial and tangential ones, $\varepsilon_{a}=\varepsilon_{t}=\varepsilon_{\parallel}$. The dielectric constant of BNNTs is described by the Lorentz model:

$\varepsilon_{m}(\omega)=\varepsilon_{\infty,m}\left(1+\frac{\omega_{LO,m}^{2}-\omega_{TO,m}^{2}}{\omega_{TO,m}^{2}-\omega^{2}-i\omega\gamma_{m}}\right).$ (11)

Here, $m=\parallel\text{，}\perp$represents the two possible electric field polarizations (in-plane or out-of- plane) directions, $\varepsilon_{\infty,m}$represent the high-frequency dielectric constants, $\gamma_{m}$represents the phonon damping, $\omega_{TO,m}$and$\omega_{LO,m}$represent the phonon frequencies of the TO and LO modes, respectively.

To describe the behaviour of the fabricated BNNT sample as a whole, we use the effective medium theory (EMT) to calculate the effective permittivity of the fabricated BNNT sample as follows ^[26, 27]^

$\varepsilon_{s}=\varepsilon_{air}\frac{\varepsilon_{hBN}\left(1+\rho\right)+\varepsilon_{air}(1-\rho)}{\varepsilon_{hBN}(1-\rho)+\varepsilon_{air}\left(1+\rho\right)},$ (12)

$\varepsilon_{l}=\varepsilon_{hBN}\rho+\varepsilon_{air}(1-\rho).$ (13)

In the equation,$\varepsilon_{air}$and$\varepsilon_{hBN}$represent the dielectric constants of air and BNNTs, respectively, and 𝜌 is the volume fraction (filling factor) of BNNTs. Since the incident light is polarized in-plane with respect to the BNNTs, we only need to consider the axial and tangential components in the calculation. Thus, the$\varepsilon_{hBN}$can be simplified as follows

$\varepsilon_{hBN}=\varepsilon_{\parallel}=\varepsilon_{\infty}\left(1+\frac{\omega_{LO}^{2}-\omega_{TO}^{2}}{\omega_{TO}^{2}-\omega^{2}-i\omega\gamma}\right),$ (14)

The parameters retain the same definitions as previously described, but pertain exclusively to the in-plane component. According to Reference,^[28]^ the parameters are given as follows: $\varepsilon_{\infty}$=4.87, $\omega_{TO}$=169.5 meV, $\omega_{LO}$=200 meV, and $\gamma$=0.86 meV. It should be also noted that the current EMT theoretical framework does not explicitly account for the fine structural details of the nanotubes. A more in-depth analysis of the bamboo-like features observed in the nanotubes and their effect on phonon polariton (PhP) propagation remains an intriguing direction for future research.

In Figure S22b, c, we present the real and imaginary parts of $\varepsilon_{s}$ and $\varepsilon_{l}$. The values of$\varepsilon_{s}$ change rapidly with the filling factor, exhibiting significant variations. In contrast, $\varepsilon_{l}$primarily shows amplitude variation and minimal displacement. The transfer matrix method (TMM) can be used to calculate the optical response (reflectance and transmittance) of multilayer structures. However, traditional TMM assumes that light propagation is coherent, leading to narrow oscillations in the calculated spectrum of the system. In fact, due to interference damping, these oscillations may not be observable. Therefore, to achieve an accurate description of the optical properties of multilayer systems, it is necessary to consider these interference damping effects.^[29]^


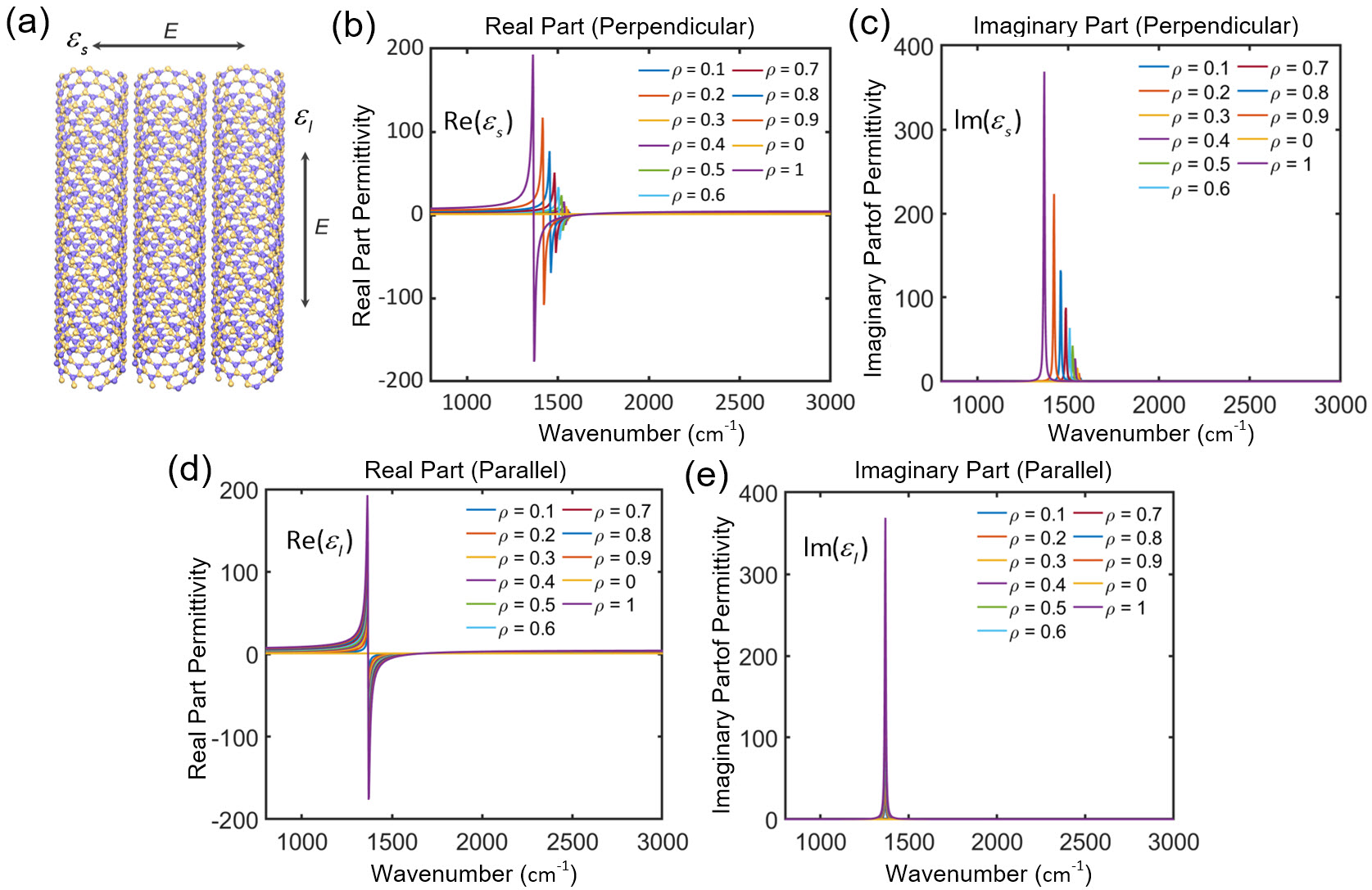


**Figure S22.** Dielectric response of BNNTs. a) The dielectric tensors of BNNTs in the perpendicular ($\varepsilon_{s}$)and parallel ($\varepsilon_{l}$) orientations. b, c) The real and imaginary parts of the effective permittivity of BNNTs for the perpendicular polarization. d, e) The real and imaginary parts of the effective permittivity of BNNTs for the parallel polarization. The legends denote different volume fractions of BNNTs in the composite material.

Long-straight BNNTs exhibit a pronounced whispering-gallery phonon polaritons (WGM-PhPs) response.^[14]^ The bamboo-like morphology of BNNTs, characterized by structural discontinuities and periodic modulation, significantly influences the propagation behavior and other responses of PhPs. Lattice distortions and interfacial defects at the bamboo-like nodes enhance the scattering and localization of PhPs, reducing their propagation distance and consequently decreasing the quality factor (Q), which may affect the linewidth of polarizers. Meanwhile, periodic nodes can induce a propagation bandgap via Bragg scattering. By modifying the dielectric constant, they can influence the dispersion relation of polaritons, enabling selective frequency tuning of PhPs. The scattering-type scanning near-field optical microscopy (s-SNOM) is an effective technique for probing PhPs, as schematically illustrated in Figure 4a. This method combines atomic force microscopy (AFM) with mid-infrared laser interferometric detection, utilizing the field enhancement at the metallic tip apex to concentrate far-field light into a nanoscale localized region, thereby enabling high spatial resolution imaging of the material's near-field optical response.^[24]^ In the experiment, a quantum cascade laser (QCL) served as the mid-infrared light source. The beam was collimated and focused onto the AFM tip using a parabolic mirror. The tip oscillates at a frequency Ω, and the scattered signal from the sample surface was demodulated at higher harmonics (e.g., 2Ω) to suppress background noise and extract the pure near-field signal.^[25, 28]^ As shown in Figure S23, spatial imaging of BNNT PhPs reveals their axial and radial propagation. At each node of the BNNTs, significant local field enhancement and mode coupling effects are observed. These findings indicate that the bamboo-like structure has a pronounced impact on PhPs. The bamboo-like structure not only serves as a scattering center for polariton propagation but also enables dynamic tuning of polariton bandgaps and localized modes through structural engineering.


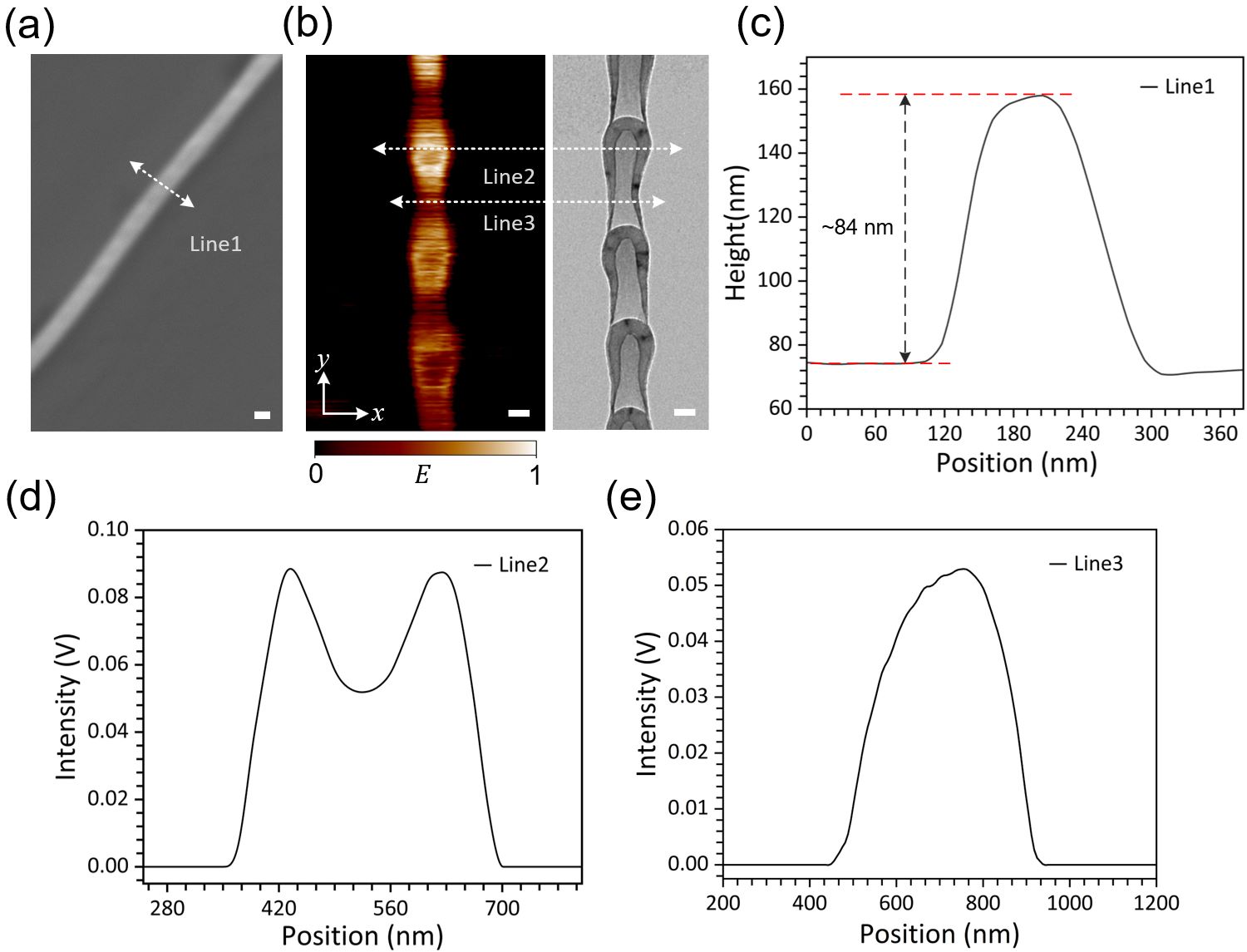


**Figure S23.** Near-field characterization and electric field distribution of a single BNNT. a) SEM image of a single BNNT. b) Near-field infrared intensity and corresponding BNNT position of a single BNNT in Figure 5. c) Measured height at position Line1 in (a), with the diameter of the BNNT inferred to be 84 nm based on its height. d) Near-field intensity distribution profile along the BNNT diameter direction at the Line2 position in (b) (node region). e) Near-field intensity distribution profile at the Line3 position (non-node region). The scale bars are 50 nm.

To validate the linear-polarized absorption of ABNNTs in the mid-infrared region and provide more reliable and detailed results, we performed calculations based on effective medium theory (EMT) and compared them with FTIR measurements using linearly polarized light. Generally, the transmittance is lower in the ranges of 790–830 cm^-1^ (RB1) and 1367–1610 cm^-1^ (RB2), which usually indicates higher absorption in these bands.^[25]^ Figure S24 shows the linearly polarized transmittance spectra from EMT calculations within the wavenumber range of 900–2600 cm^-1^, including results for parallel and perpendicular transmittance at three wavenumbers: 2469 cm^-1^, 1527 cm^-1^, and 1116 cm^-1^. It can be observed that ABNNTs exhibit a higher absorption rate for parallel-polarized infrared light compared to that for perpendicular-polarized light，which is consistent with the FTIR polarization test results at three wavelengths shown in Figure 4d-f. Specifically, in Figure 4e, f, the transmittance of parallel-polarized light at 2469 cm^-1^ and 1116 cm^-1^ is approximately 41.33% and 56.20%, respectively. In Figure 4d, the transmittance of parallel-polarized light at 1527 cm^-1^ is about 9.64%. The relationship of polarization-dependent absorption rates follows: A (1527 cm^-1^) > A (2469 cm^-1^) > A (1116 cm^-1^).


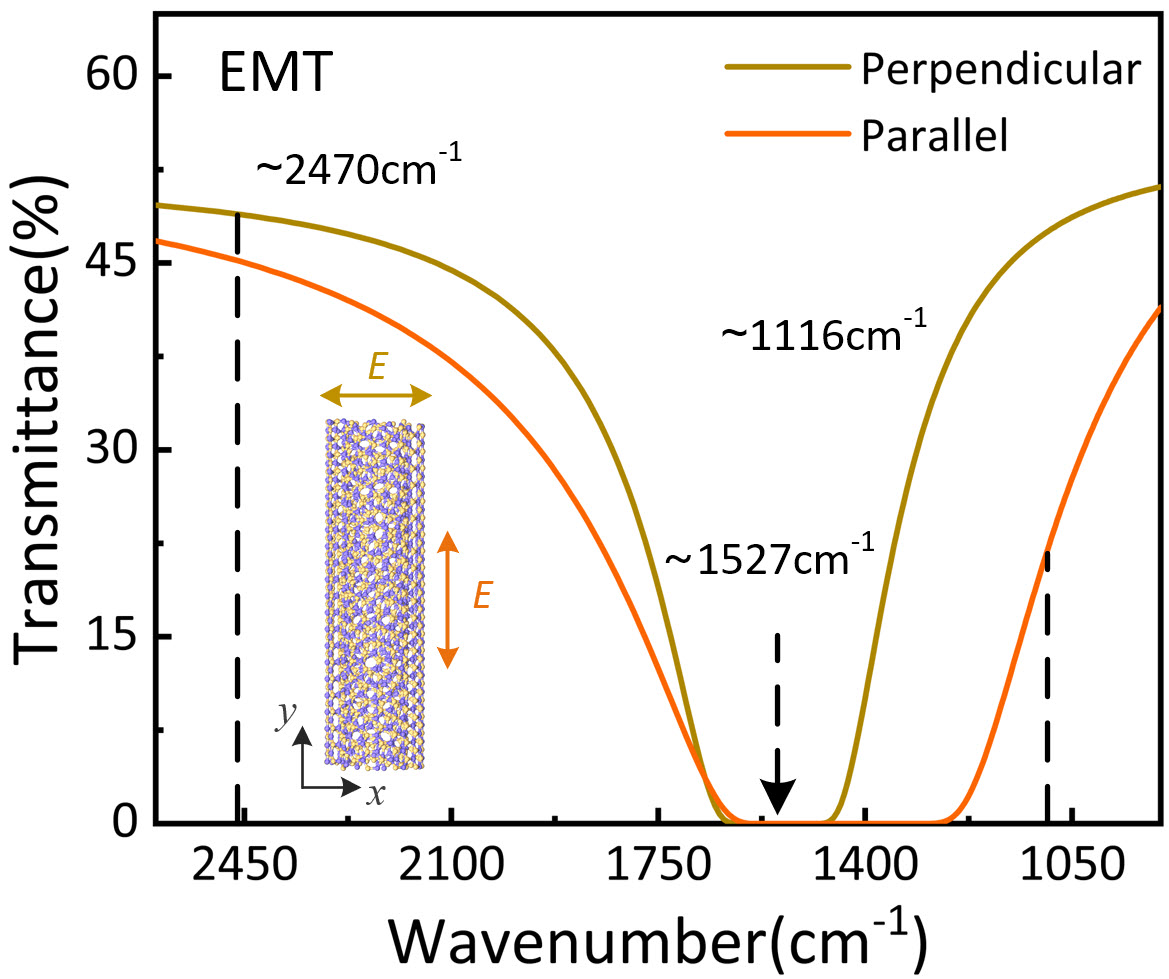


**Figure S24.** EMT-simulated polarization-dependent FTIR calculations for BNNTs, including the simulated transmittance results for parallel and perpendicular polarization at 2469 cm^-1^, 1527 cm^-1^, and 1116 cm^-1^.


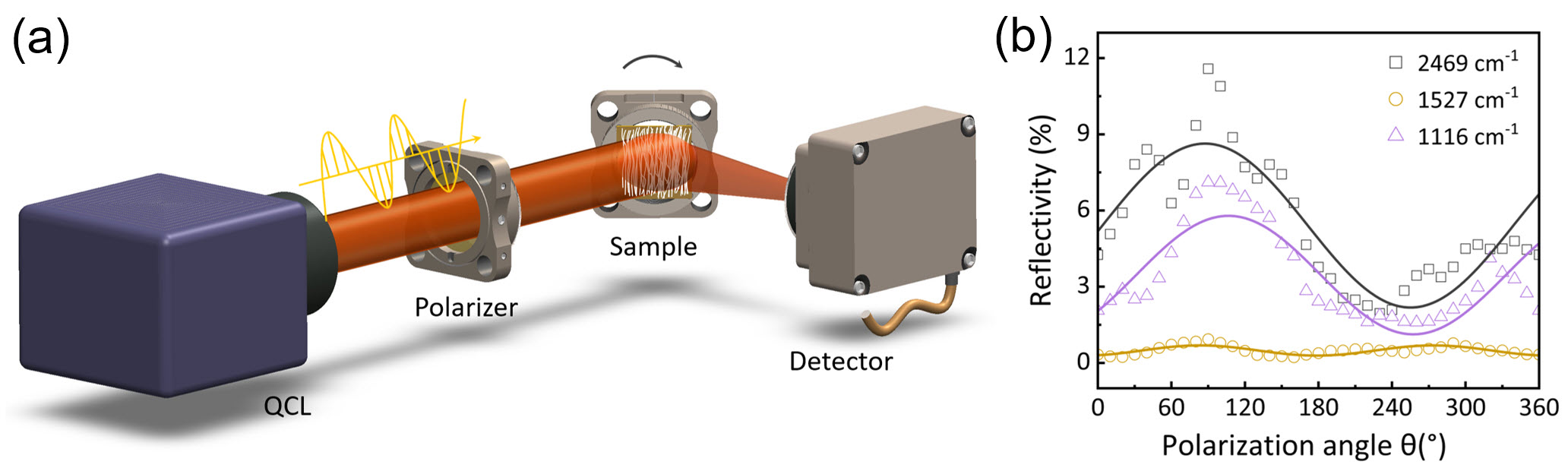


**Figure S25.** a) Schematic diagram of the polarization-sensitive optical reflection measurement system. b) Polarization-dependent reflection of ABNNTs under three types of polarized light illumination.

To further discuss the linear polarization sensitivity of BNNTs, we performed specular reflection measurements under oblique incidence. Figure S26a shows the optical path of the linearly polarized oblique incidence reflection system, with polarization angles ranging from 0° to 360°, incremented by 10°. Figure S26b, c show the relationship between the oblique incidence reflection of polarized light and the polarization angle. It can be observed that ABNNTs exhibit sensitivity to linearly polarized light at 2469 cm^-1^, 1527 cm^-1^, and 1116 cm^-1^. The grazing-incidence transmittance relationship follows T (1116 cm^-1^) > T (2469 cm^-1^) > T (1527 cm^-1^). Within the polarization angle range of 0° to 360°, the grazing-incidence reflectance of polarized light at the three wavenumbers exhibits a periodic double-peak profile. It was demonstrated that the reflection characteristics of BNNTs exhibit wavelength dependence, indicating that the optical properties of BNNTs are closely related to the wavelength. This result suggests that BNNTs have potential applications in the optical field, particularly in the infrared spectral range.


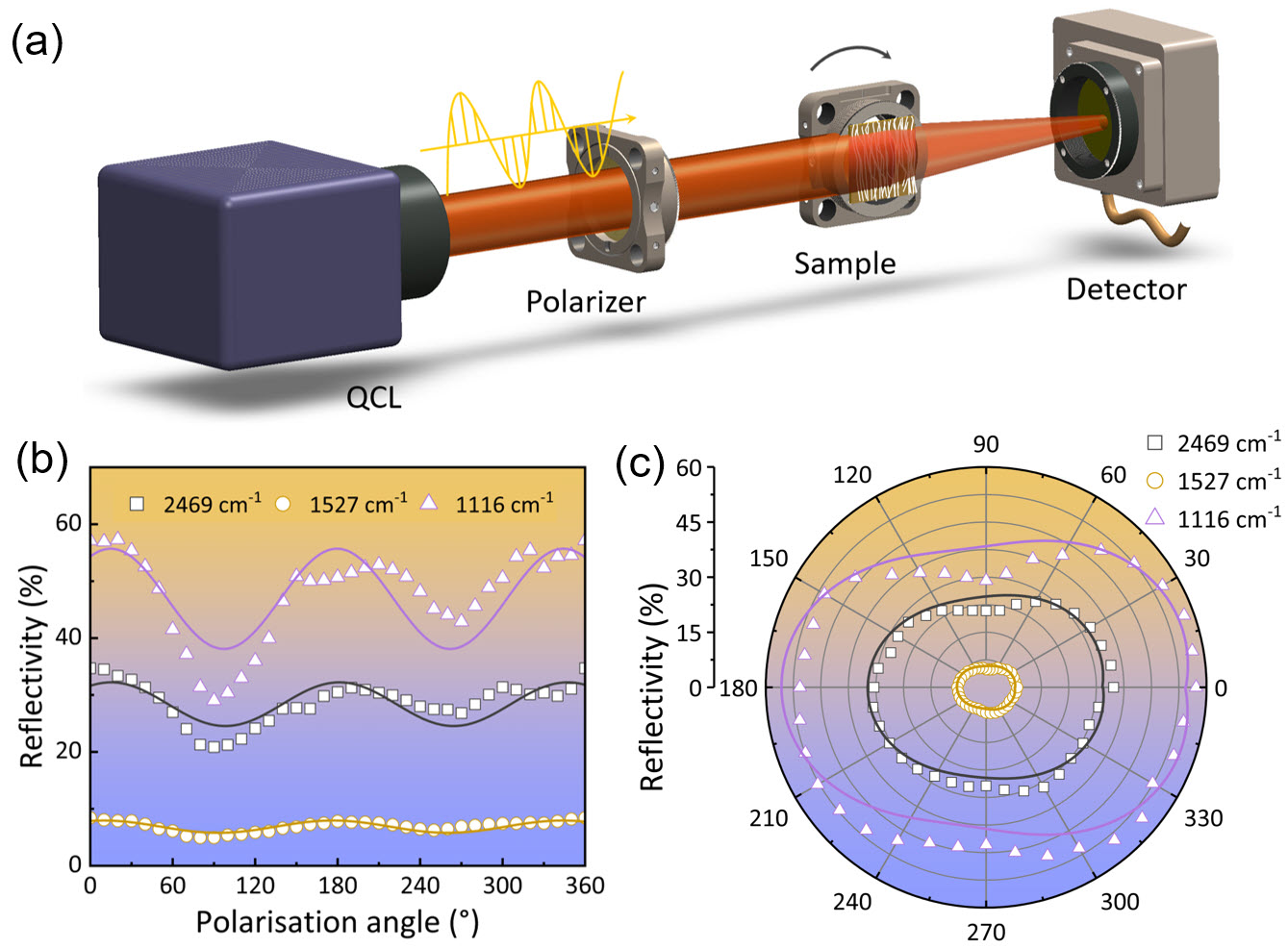


**Figure S26.** Specular reflection polarization-sensitive optical detection of ABNNTs under oblique incidence. a) Optical path of the linearly polarized oblique incidence reflection system. b) Polarization-dependent grazing-incidence reflection of BNNTs under linearly polarized light at three different wavenumbers, normalized and fitted using a sine function. c) Polar plot of polarization-sensitive absorption for linearly polarized light at three wavenumbers.

The diameter of BNNTs can be tuned by adjusting synthesis parameters such as catalyst selection, precursor concentration, growth temperature, and pressure. Variations in diameter influence the volume fraction of BNNTs within composite materials, thereby affecting their effective dielectric constant, as shown in Figure S22. It is anticipated that a reduction in BNNT diameter leads to a significant increase in linear dichroism and polarization ratio, while an increase in diameter reduces polarization selectivity. Therefore, precise control over BNNT diameter provides a feasible strategy to optimize their polarization sensitivity and linear dichroism, offering a promising route for performance tuning in infrared polarizer applications.

**Table S1.** Comparison of Anisotropy in Different Materials.

| Time | Publication | Materials | Wavelength | Anisotropy Ratio | Deformation stability |
| --- | --- | --- | --- | --- | --- |
| 2017 | J. Am. Chem. Soc.  **139**, 14976 (2017) | GeSe | 532/638 / 808 nm | 1.09/1.44/2.16 | NO |
| 2018 | ACS Nano **12**,  8807 (2018) | TlSe | 633nm | 2.65 | NO |
| 2021 | Adv. Mater. **34**,  2105665 (2022) | 2,6-diphenyl anthracene (DPA) | 450nm | 1.9 | NO |
| 2024 | Nano Lett. **24**,  7716 (2024) | WSe_2_  (Self-Rolled-Up) | 638/808 nm | 2.06/1.96 | NO |
| 2024 | Nat. Photon. **18**,  1176 (2024) | SWCNTs | 1660/2200 nm | 0.58 | NO |
| **2024** | **Our work** | **BNNTs** | **1.55/4.05/6.55/8.96μm** | **4.53/1.54/1.04/4.34** | **Yes** |

**References**

1. Zhao, T.; Guo, J.; Li, T.; Wang, Z.; Peng, M.; Zhong, F.; Chen, Y.; Yu, Y.; Xu, T.; Xie, R.; Gao, P.; Wang, X.; Hu, W. Substrate Engineering for Wafer-Scale Two-Dimensional Material Growth: Strategies, Mechanisms, and Perspectives. Chemical Society Reviews. Royal Society of Chemistry February 6, 2023, pp 1650–1671. <https://doi.org/10.1039/d2cs00657j>.
2. Liu, C.; Wang, L.; Qi, J.; Liu, K. Designed Growth of Large‐Size 2D Single Crystals. Advanced Materials 2020, 32 (19). <https://doi.org/10.1002/adma.202000046>.
3. Tang, L.; Tan, J.; Nong, H.; Liu, B.; Cheng, H.-M. Chemical Vapor Deposition Growth of Two-Dimensional Compound Materials: Controllability, Material Quality, and Growth Mechanism. Acc Mater Res 2021, 2 (1), 36–47. <https://doi.org/10.1021/accountsmr.0c00063>.
4. Zhi, C.; Bando, Y.; Tang, C.; Golberg, D. Boron Nitride Nanotubes. In Materials Science and Engineering R: Reports; 2010; Vol. 70, pp 92–111. <https://doi.org/10.1016/j.mser.2010.06.004>.
5. Li, L.; Li, L. H.; Chen, Y.; Dai, X. J.; Lamb, P. R.; Cheng, B.; Lin, M.; Liu, X. High‐Quality Boron Nitride Nanoribbons: Unzipping during Nanotube Synthesis. Angewandte Chemie International Edition 2013, 52 (15), 4212–4216. <https://doi.org/10.1002/anie.201209597>.
6. Zhang, L.; Dong, J.; Ding, F. Strategies, Status, and Challenges in Wafer Scale Single Crystalline Two-Dimensional Materials Synthesis. Chem Rev 2021, 121 (11), 6321–6372. <https://doi.org/10.1021/acs.chemrev.0c01191>.
7. Wan, Y.; Fu, J.-H.; Chuu, C.-P.; Tung, V.; Shi, Y.; Li, L.-J. Wafer-Scale Single-Orientation 2D Layers by Atomic Edge-Guided Epitaxial Growth. Chem Soc Rev 2022, 51 (3), 803–811. <https://doi.org/10.1039/D1CS00264C>.
8. Wang, L.; Li, T.; Long, X.; Wang, X.; Xu, Y.; Yao, Y. Bimetallic Catalytic Growth of Boron Nitride Nanotubes. Nanoscale 2017, 9 (5), 1816–1819. <https://doi.org/10.1039/C6NR08623C>.
9. Chen, Y.; Chadderton, L. T.; Gerald, J. F.; Williams, J. S. A Solid-State Process for Formation of Boron Nitride Nanotubes. Appl Phys Lett 1999, 74 (20), 2960–2962. <https://doi.org/10.1063/1.123979>.
10. Lin, C. R.; Su, C. H.; Hung, C. H.; Chang, C. Y.; Stobinski, L. Characterization of Bamboo-like CNTs Prepared Using Sol–Gel Catalyst. Diam Relat Mater 2005, 14 (3–7), 794–797. <https://doi.org/10.1016/j.diamond.2004.12.005>.
11. Lu, G.; Wu, T.; Yuan, Q.; Wang, H.; Wang, H.; Ding, F.; Xie, X.; Jiang, M. Synthesis of Large Single-Crystal Hexagonal Boron Nitride Grains on Cu–Ni Alloy. Nat Commun 2015, 6 (1), 6160. <https://doi.org/10.1038/ncomms7160>.
12. Lee, S.-H.; Kim, M. J.; Ahn, S.; Koh, B. Purification of Boron Nitride Nanotubes Enhances Biological Application Properties. Int J Mol Sci 2020, 21 (4), 1529. <https://doi.org/10.3390/ijms21041529>.
13. Amin, M. S.; Molin, T. E.; Tampubolon, C.; Kranbuehl, D. E.; Schniepp, H. C. Boron Nitride Nanotube Impurity Detection and Purity Verification. Chemistry of Materials 2020, 32 (21), 9090–9097. <https://doi.org/10.1021/acs.chemmater.0c03609>.
14. Phillips, C.; Gilburd, L.; Xu, X. G.; Walker, G. C. Surface and Volume Phonon Polaritons in Boron Nitride Nanotubes. J Phys Chem Lett 2019, 10 (17), 4851–4856. <https://doi.org/10.1021/acs.jpclett.9b01829>.
15. Maselugbo, A. O.; Harrison, H. B.; Alston, J. R. Boron Nitride Nanotubes: A Review of Recent Progress on Purification Methods and Techniques. J Mater Res 2022, 37 (24), 4438–4458. <https://doi.org/10.1557/s43578-022-00672-5>.
16. Chen, H.; Chen, Y.; Yu, J.; Williams, J. S. Purification of Boron Nitride Nanotubes. Chem Phys Lett 2006, 425 (4–6), 315–319. <https://doi.org/10.1016/j.cplett.2006.05.058>.
17. Ikazaki, F.; Ohshima, S.; Uchida, K.; Kuriki, Y.; Hayakawa, H.; Yumura, M.; Takahashi, K.; Tojima, K. Chemical Purification of Carbon Nanotubes by Use of Graphite Intercalation Compounds. Carbon N Y 1994, 32 (8), 1539–1542. <https://doi.org/10.1016/0008-6223(94)90152-X>.
18. Rinzler, A. G.; Liu, J.; Dai, H.; Nikolaev, P.; Huffman, C. B.; Rodríguez-Macías, F. J.; Boul, P. J.; Lu, A. H.; Heymann, D.; Colbert, D. T.; Lee, R. S.; Fischer, J. E.; Rao, A. M.; Eklund, P. C.; Smalley, R. E. Large-Scale Purification of Single-Wall Carbon Nanotubes: Process, Product, and Characterization. Appl Phys A Mater Sci Process 1998, 67 (1), 29–37. <https://doi.org/10.1007/s003390050734>.
19. Ko, J.; Kim, H. M.; Moon, S. Y.; Ahn, S.; Im, S. G.; Joo, Y. Highly Pure, Length-Sorted Boron Nitride Nanotubes by Gel Column Chromatography. Chemistry of Materials 2021, 33 (12), 4723–4732. <https://doi.org/10.1021/acs.chemmater.1c01165>.
20. Amin, M. S.; Atwater, B.; Pike, R. D.; Williamson, K. E.; Kranbuehl, D. E.; Schniepp, H. C. High-Purity Boron Nitride Nanotubes via High-Yield Hydrocarbon Solvent Processing. Chemistry of Materials 2019, 31 (20), 8351–8357. <https://doi.org/10.1021/acs.chemmater.9b01713>.
21. Harrison, H.; Lamb, J. T.; Nowlin, K. S.; Guenthner, A. J.; Ghiassi, K. B.; Kelkar, A. D.; Alston, J. R. Quantification of Hexagonal Boron Nitride Impurities in Boron Nitride Nanotubes via FTIR Spectroscopy. Nanoscale Adv 2019, 1 (5), 1693–1701. <https://doi.org/10.1039/C8NA00251G>.
22. Tay, R. Y.; Li, H.; Tsang, S. H.; Jing, L.; Tan, D.; Wei, M.; Teo, E. H. T. Facile Synthesis of Millimeter-Scale Vertically Aligned Boron Nitride Nanotube Forests by Template-Assisted Chemical Vapor Deposition. Chemistry of Materials 2015, 27 (20), 7156–7163. <https://doi.org/10.1021/acs.chemmater.5b03300>.
23. Liu, M.; Wang, S.; Zheng, Y.; Takeuchi, M.; Inoue, T.; Xiang, R.; Maruyama, S. Synthesis of Vertically Aligned Boron Nitride Nanotubes with a Template of Single-Walled Carbon Nanotubes. J Mater Res 2022, 37 (24), 4428–4437. <https://doi.org/10.1557/s43578-022-00759-z>.
24. Low, T.; Chaves, A.; Caldwell, J. D.; Kumar, A.; Fang, N. X.; Avouris, P.; Heinz, T. F.; Guinea, F.; Martin-Moreno, L.; Koppens, F. Polaritons in Layered Two-Dimensional Materials. Nat Mater 2017, 16 (2), 182–194. <https://doi.org/10.1038/nmat4792>.
25. Dai, S.; Fang, W.; Rivera, N.; Stehle, Y.; Jiang, B.; Shen, J.; Tay, R. Y.; Ciccarino, C. J.; Ma, Q.; Rodan‐Legrain, D.; Jarillo‐Herrero, P.; Teo, E. H. T.; Fogler, M. M.; Narang, P.; Kong, J.; Basov, D. N. Phonon Polaritons in Monolayers of Hexagonal Boron Nitride. Advanced Materials 2019, 31 (37). <https://doi.org/10.1002/adma.201806603>.
26. Starko-Bowes, R.; Atkinson, J.; Newman, W.; Hu, H.; Kallos, T.; Palikaras, G.; Fedosejevs, R.; Pramanik, S.; Jacob, Z. Optical Characterization of Epsilon-near-Zero, Epsilon-near-Pole, and Hyperbolic Response in Nanowire Metamaterials. Journal of the Optical Society of America B 2015, 32 (10), 2074. <https://doi.org/10.1364/JOSAB.32.002074>.
27. Markel, V. A. Introduction to the Maxwell Garnett Approximation: Tutorial. Journal of the Optical Society of America A 2016, 33 (7), 1244. <https://doi.org/10.1364/JOSAA.33.001244>
28. Guo, X.; Li, N.; Yang, X.; Qi, R.; Wu, C.; Shi, R.; Li, Y.; Huang, Y.; García de Abajo, F. J.; Wang, E.-G.; Gao, P.; Dai, Q. Hyperbolic Whispering-Gallery Phonon Polaritons in Boron Nitride Nanotubes. Nat Nanotechnol 2023, 18 (5), 529–534. <https://doi.org/10.1038/s41565-023-01324-3>.
29. Centurioni, E. Generalized Matrix Method for Calculation of Internal Light Energy Flux in Mixed Coherent and Incoherent Multilayers. Appl Opt 2005, 44 (35), 7532. <https://doi.org/10.1364/AO.44.007532>.
